# Supplementary material for: Cross ionization mode chemical similarity prediction between tandem mass spectra in metabolomics
Source: Nat Commun. 2026 Feb 7;17:2483. doi: 10.1038/s41467-026-69083-y (PMC12992594; doi:10.1038/s41467-026-69083-y)
Supplement: Supplementary file 1 — Supplementary Information [file 41467_2026_69083_MOESM1_ESM.pdf]

Supplementary Information belonging to:

Cross ionization mode chemical similarity prediction between tandem mass spectra in metabolomics

Authors:

Niek F. de Jonge[1,#], Elena Chekmeneva[2], Robin Schmid[3,4], David Joas[5], Lem-Joe Truong[5], Justin J.J. van der Hooft[1,6,#,*], Florian Huber[5,#,*]

[1] Bioinformatics Group, Wageningen University & Research, 6708 PB, Wageningen, the Netherlands
[2] The National Phenome Centre, Section of Bioanalytical Chemistry, Division of Systems Medicine, Department of Metabolism, Digestion and Reproduction, Faculty of Medicine, Imperial College London, Hammersmith Hospital Campus, London, W12 0NN, United Kingdom
[3] Institute of Organic Chemistry and Biochemistry of the Czech Academy of Sciences, Prague, Czechia
[4] mzio GmbH, Bremen, Germany
[5] Centre for Digitalisation and Digitality (ZDD), University of Applied Sciences Düsseldorf, Düsseldorf, Germany
[6] Department of Biochemistry, University of Johannesburg, Auckland Park, Johannesburg, 2006, South Africa

# Corresponding authors: niek.dejonge@wur.nl, justin.vanderhooft@wur.nl, florian.huber@hs-duesseldorf.de
* These authors jointly supervised this work.

Contents

Supplementary Tables 2
Supplementary Note 1. Hyperparameter optimization 3
Supplementary Note 2. Pair sampling optimization 7
Supplementary Note 3. Comparison to MS2DeepScore 0.2.0 model 13
Supplementary Note 4: Comparison to model trained on single ionization mode 15
Supplementary Note 5. Comparison to modified cosine score 17
Supplementary Note 6. Embedding Evaluator 19
Supplementary Note 7. Detailed analysis of MS2DeepScore model 21
Supplementary Note 8. Human blood plasma case study 23
Supplementary Note 9. Spectrum comparison plots 24
Supplementary Note 10: Benchmarking specific use cases 25
Supplementary Methods 30
Supplementary References 32

Supplementary Tables

Supplementary Table 1: Case study annotations. **Levels of confidence in the assignment of the identified metabolites according to the Metabolomics Standards Initiative[1]. Level 1 annotations are confirmed with an in-house standard. Level 2 annotations are confirmed by comparison to a standard in the NIST 23 (2023) mass spectral library[2]. Level 3 annotations are putative annotation based on the MS/MS spectrum interpretation. Some metabolite annotations are repeated, since for some metabolites a mass spectrum was recorded multiple times in different fractions. **Spectra with the same cluster number are connected in the molecular network in Figure 3a.*

m/z	**RT in min**	**ionization mode**	**Name**	**Confidence level of annotation***	**Cluster****
181.0379	1.83	negative	1-Methyluric acid	1	1
181.0366	1.82	negative	1-Methyluric acid	1	1
181.0385	1.83	negative	1-Methyluric acid	1	1
195.0569	2.29	negative	1,3-Dimethyluric acid	2	1
195.0524	2.61	negative	1,7-Dimethyluric acid	1	1
195.0525	2.29	negative	1,3-Dimethyluric acid	2	1
181.0727	2.86	positive	Theophylline	1	1
181.0725	1.11	positive	5-Acetylamino-6-amino-3-methyluracil	2	1
195.091	3.61	positive	Caffeine	1	1
195.0993	3.60	positive	Caffeine	1	1
181.074	1.11	positive	5-Acetylamino-6-amino-3-methyluracil	2	1
181.0727	2.86	positive	Theophylline	1	1
195.091	3.61	positive	Caffeine	1	1
195.0993	3.60	positive	Caffeine	1	1
181.0725	1.11	positive	5-Acetylamino-6-amino-3-methyluracil	2	1
181.074	1.11	positive	5-Acetylamino-6-amino-3-methyluracil	2	1
158.0820	3.01	negative	N-Acetyl-L-valine	3	2
160.0395	5.07	negative	Indole-3-carboxylic acid	2	2
190.0499	3.20	positive	Kynurenic acid	1	2
162.0557	4.11	positive	2,8-Quinolinediol	2	2
162.0564	4.11	positive	2,8-Quinolinediol	2	2
162.0538	5.15	positive	Indole-3-carboxylic acid	2	2
190.0520	3.21	positive	Kynurenic acid	1	2
190.0499	3.20	positive	Kynurenic acid	1	2
162.0557	4.11	positive	2,8-Quinolinediol	2	2
162.0564	4.11	positive	2,8-Quinolinediol	2	2
162.0538	5.15	positive	Indole-3-carboxylic acid	2	2
190.0468	3.11	positive	Kynurenic acid	1	2
190.0468	3.11	positive	Kynurenic acid	1	2
190.0498	3.15	positive	Kynurenic acid	1	2
190.0498	3.15	positive	Kynurenic acid	1	2
190.0520	3.21	positive	Kynurenic acid	1	2
151.0349	2.95	positive	Homogentisic acid	2	3
151.0349	2.95	positive	Homogentisic acid	2	3
151.0351	1.33	negative	3,4-Dihydroxyphenylglycol	2	3
407.2797	9.72	negative	Cholic acid	1	UMAP
355.2637	9.73	positive	Cholic acid	1	UMAP

Supplementary Notes

Supplementary Note 1. Hyperparameter optimization

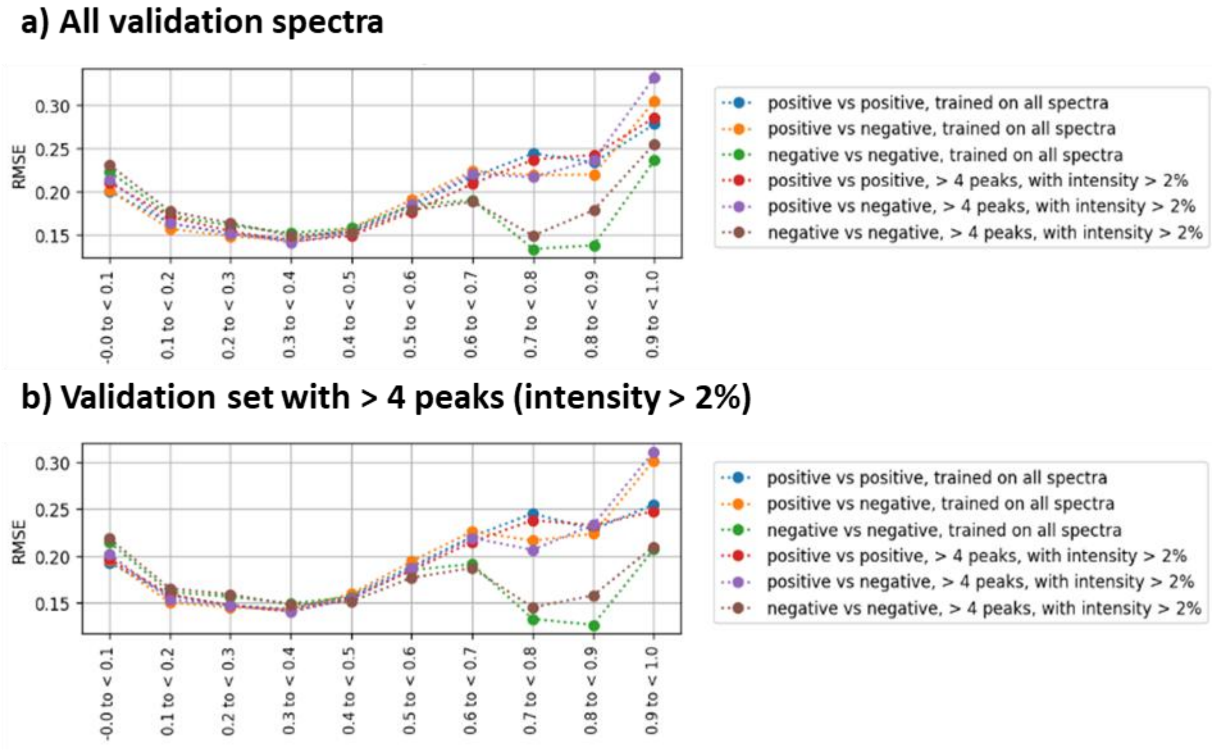

Supplementary Figure 1: Average RMSE per bin for model trained with different filtering of training spectra. *For one of the models the training spectra only contained spectra that have at least 5 peaks at intensity >2%. The other model was trained on all spectra. The model trained on all spectra is used in the rest of the paper, since it resulted in best overall performance. Both models were trained with identical settings. The raw data and notebook required to reproduce this figure is available in the Source Data file.* ***a)*** *Both models were validated with the standard validation set, containing all spectra.* ***b)*** *Both models were validated with the validation set only containing spectra with at least 5 peaks at intensity >2%.*

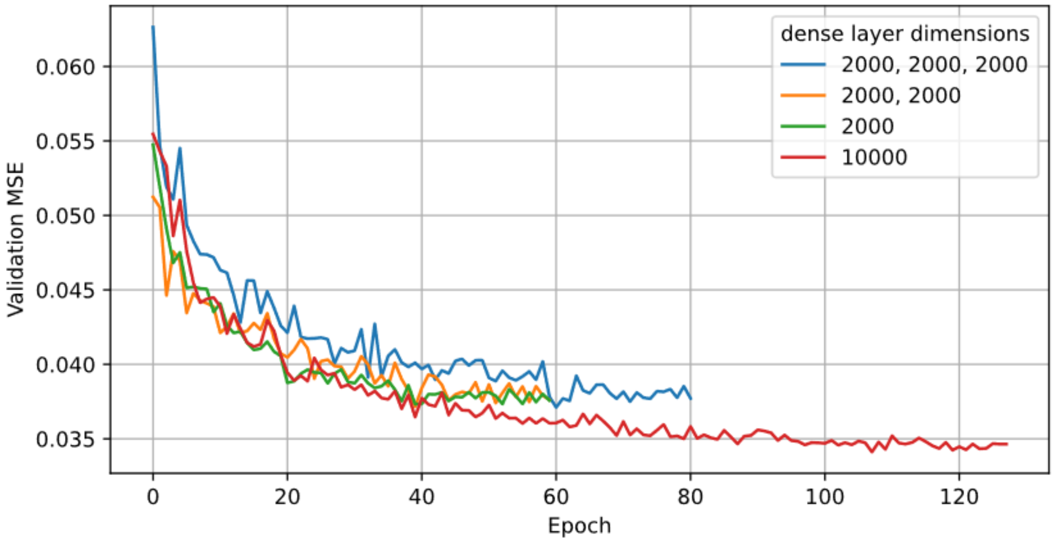

Supplementary Figure 2: Models trained with varying model architectures. *The model architecture was varied to select a suitable model architecture. The models all used the same hyperparameters as the final model, except for the number of layers and dimensions of these layers. The input layer always consisted of 10000 bins with a width of 0.1 Da and a final embedding size of 500. The legend shows the dense layers used between the input layer and the predicted embedding, e.g., 2000, 2000, 2000 is a dense neural network with 3 fully connected layers, each with 2000 nodes. The validation MSE was calculated over the spectra in the validation test set. To calculate the validation MSE, the pairs were binned in 10 equal Tanimoto score bins between 0 and 1, the MSE was calculated per bin and the average was taken over the 10 bins. The raw data and notebook required to reproduce this figure is available in the Source Data file.*

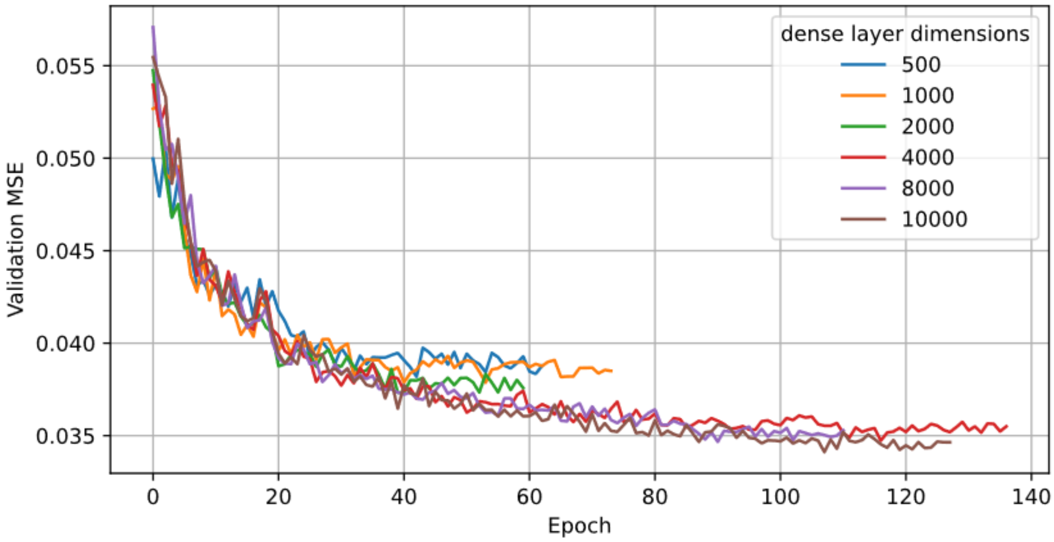

Supplementary **Figure 3: Models trained with a single dense neural network layer varying the dense layer dimension.** *The model architecture was varied to select a suitable model architecture.* The models all used a single dense layer between the input data and the predicted embedding. The dimension of this single layer was varied. *The validation MSE was calculated over the spectra in the validation test set.* To calculate the validation MSE, *the pairs were binned in 10 equal Tanimoto score bins between 0 and 1, the MSE was calculated per bin and the average was taken over the 10 bins. The raw data and notebook required to reproduce this figure is available in the Source Data file.*

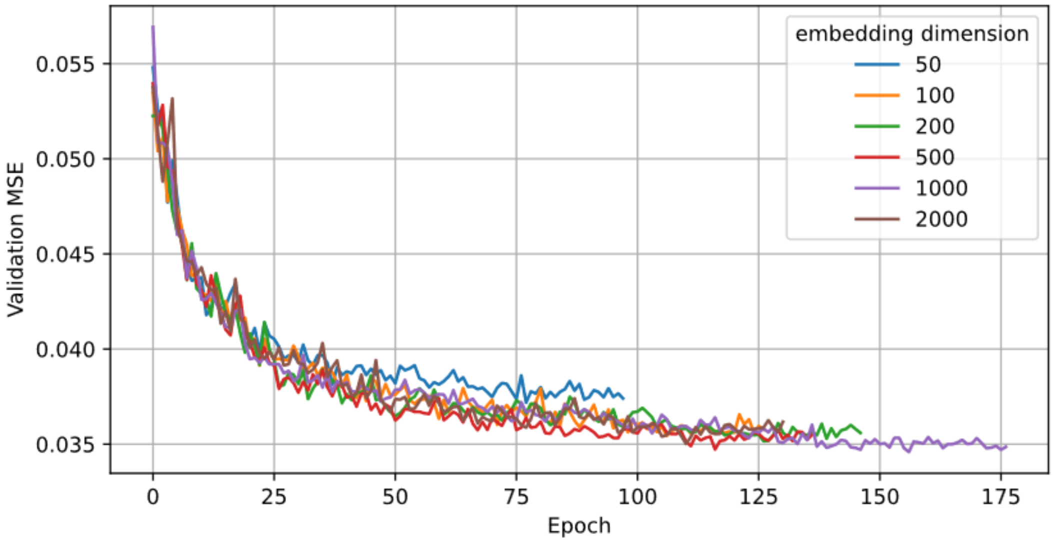

Supplementary Figure 4: Average MSE per bin for different embedding dimensions. *The models all used the same hyperparameter settings, except for the embedding size used. The validation MSE was calculated over the spectra in the validation test set. The pairs were binned in 10 equal Tanimoto score bins between 0 and 1, the MSE was calculated per bin and the average was taken over the 10 bins. The raw data and notebook required to reproduce this figure is available in the Source Data file.*

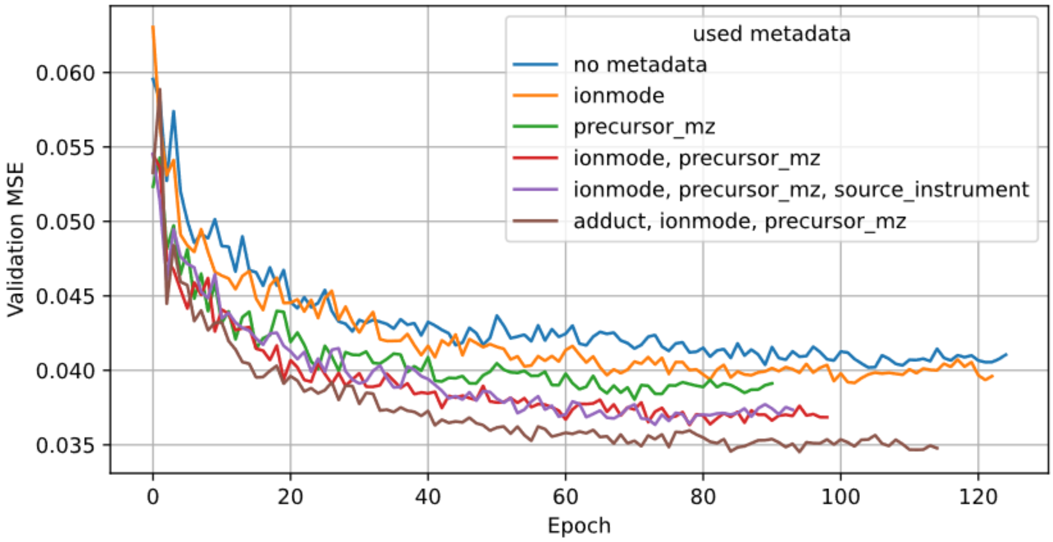

Supplementary Figure 5: Average MSE per bin for dual-ionization mode model trained with different metadata input. *The models all used the same hyperparameter settings, except for the additional metadata used. An embedding size of 500 and a single layer of size 2000 were used. The validation MSE was calculated over the spectra in the validation test set. The pairs were binned in 10 equal Tanimoto score bins between 0 and 1, the MSE was calculated per bin and the average was taken over the 10 bins. The raw data and notebook required to reproduce this figure is available in the Source Data file.*

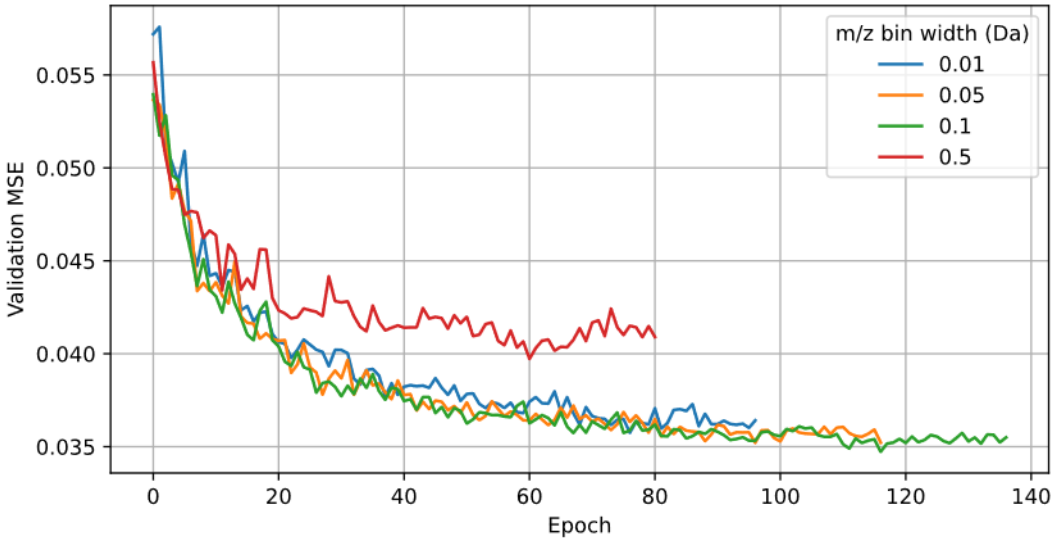

Supplementary Figure 6: Average MSE per bin for dual-ionization mode model trained with different m/z bin widths. *The models all used the same hyperparameter settings, except for the loss function used. The validation MSE was calculated over the spectra in the validation test set. The pairs were binned in 10 equal Tanimoto score bins between 0 and 1, the MSE was calculated per bin and the average is taken over the 10 bins. The raw data and notebook required to reproduce this figure is available in the Source Data file.*

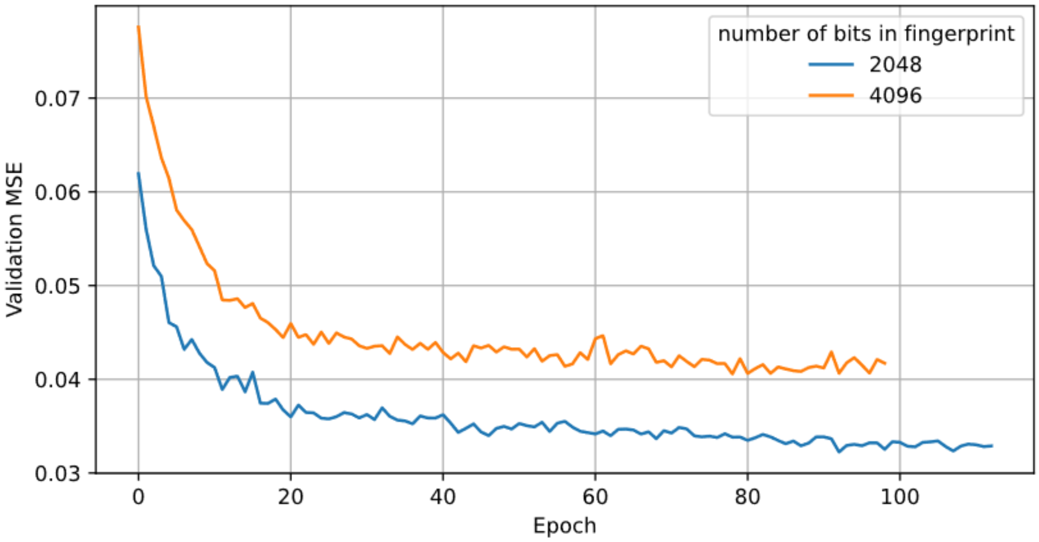

Supplementary Figure 7: Average MSE per bin model trained to predict similarity based on fingerprints with different numbers of bits. *The models both used the same hyperparameter settings, except for the number of bits used for the Daylight fingerprint. The validation MSE was calculated over the spectra in the validation test set. The pairs were binned in 10 equal Tanimoto score bins between 0 and 1, the MSE was calculated per bin, and the average was taken over the 10 bins. The raw data and notebook required to reproduce this figure is available in the Source Data file.*

Supplementary Note 2. Pair sampling optimization

Sampling molecule pairs is a crucial step in optimizing model training. Sampling random pairs would result in a very large fraction of pairs having low similarity, since most molecule pairs have a low similarity score. To ensure a more equal sampling distribution, we sample equally from 10 equally spaced bins between 0 and 1. In all the sampling algorithm optimization tests in this section, except Supplementary Figure 13, we used settings resulting in sampling each molecule on average 100 times.

Balanced molecule sampling

Randomly sampling an equal number of pairs from each Tanimoto bin is possible, but results in unequal sampling of molecules, see Supplementary Figure 8a. This is not an efficient way of using the diversity in the training data and might result in inferior generalization.

In our sampling algorithm, we counteract this by tracking the molecule sampling frequency. During sampling, the least sampled molecule is picked, followed by sampling the least frequently sampled molecule that has a pair in this bin. This still results in some disbalance in molecule frequency, since for the second molecule of a pair there is sometimes only one or a few already frequently sampled options. To counteract this, we have implemented a maximum sampling frequency. If the maximum sampling frequency is reached the molecule will not be sampled even if it is the only available pair for the least sampled molecule in that bin. To enable setting a maximum molecule sampling frequency that is close to the average molecule frequency we need to allow resampling of pairs. Otherwise, it may result in an insufficient number of molecule pairs available to sample equally in each bin. Combined this results in both balanced sampling across Tanimoto bins and little variation in molecule sampling frequency.

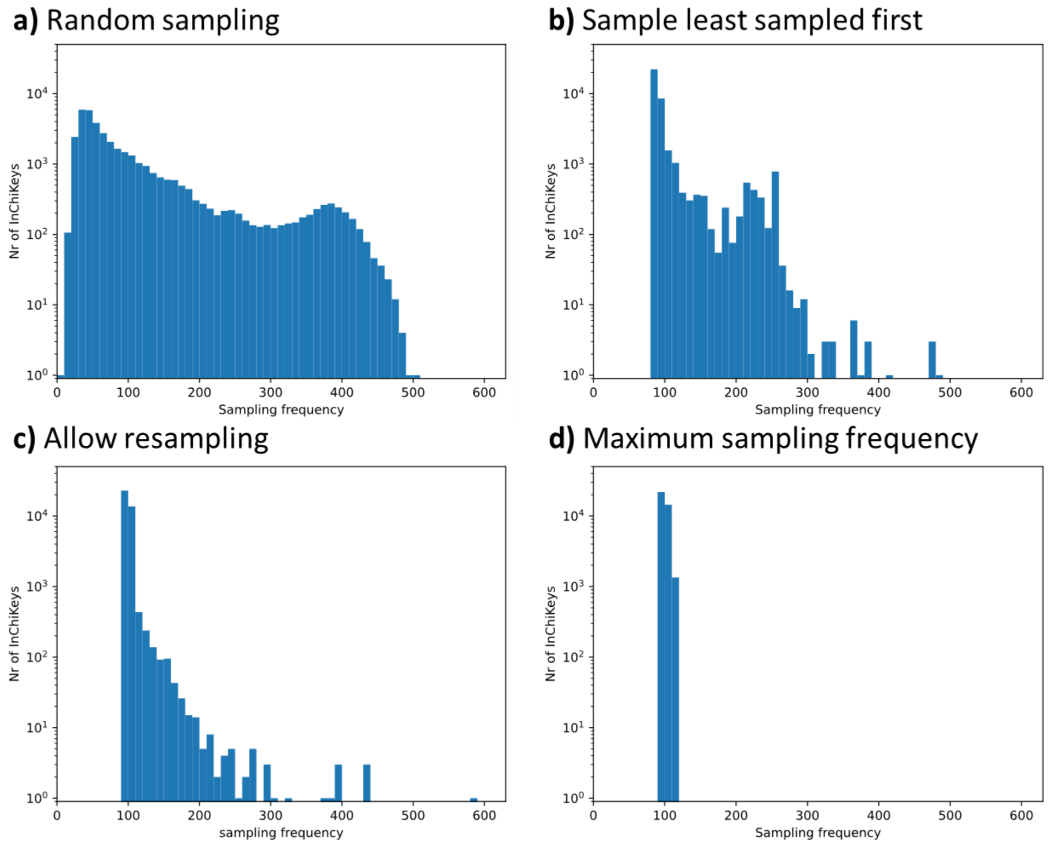

Supplementary Figure 8: The molecule sampling frequency for different sampling algorithms. *The different sampling algorithms are all tested in the training set. The sampling frequency is the number of times a unique InChIKey is sampled. The raw data and notebook required to reproduce this figure is available in the Source Data file.* ***a)*** *Random sampled an equal number of pairs from each bin.* ***b)*** *Sampled least frequently sampled molecules first.* ***c)*** *Sampled least frequently sampled molecules first, but allowed resampling of pairs that have already been sampled.* ***d)*** *Sampled least frequent molecules first, allowed resampling of pairs and set a maximum of molecule frequency at 110.*

Balanced score distribution per molecule

The sampling algorithm mentioned above results in perfect balance over the Tanimoto bins and almost equal sampling per molecule. However, per molecule the pair distribution is not balanced. For some pairs, mainly pairs with low Tanimoto scores are sampled, and for others mainly pairs with high Tanimoto scores are sampled. This imbalance can be due to biases in our training data, as some chemical classes might be more common in our dataset, while others might be unique molecules without similarity in this training set. It is not recommended to train a model that is able to use these potential biases during training. The risk is that the model learns that a molecule always has a low similarity during training. A model that uses these biases will not generalize well when predicting a highly chemically similar pair during deployment.

Ideally, each molecule would be sampled equally from each Tanimoto score bin. However, this is not possible, since many molecules have no pair available in some of the Tanimoto bins. These cases are unequally distributed over the bins, see Supplementary Figure 9. All molecules have at least one pair in the bin between 0.9-1.0, since all molecules have a Tanimoto score of 1, when comparing to itself.

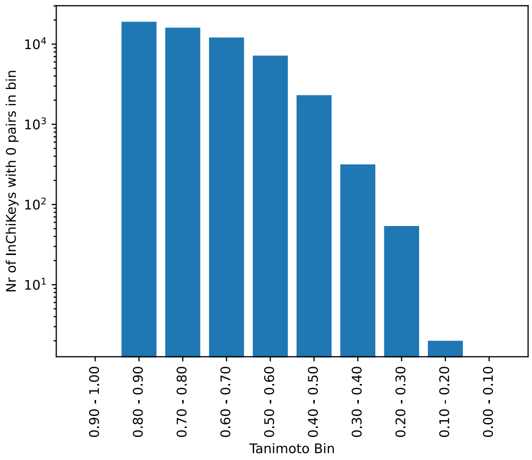

Supplementary Figure 9: Molecule frequency of molecules with 0 pairs in each bin. *The number of unique InChIKeys with 0 pairs in each Tanimoto bin for the training spectra. The raw data and notebook required to reproduce this figure is available in the Source Data file.*

Optimize bin order

The pairs are sampled per bin, molecules that are under sampled, since they don't have any pairs in a bin can be compensated in later bins. To make sure that every pair can actually be compensated by bins sampled later, the best approach is to start in the bin with the largest number of molecules with 0 pairs. This leaves more options for resampling in the following bins. However, most of the missing molecules in the bins 0.6-0.9 would in that case be oversampled in the bins 0.6-0.4, resulting in these molecules having a low average Tanimoto score. Ideally molecules with no pairs in bins are compensated in bins with similar Tanimoto scores, e.g. compensating missing pairs between 0.8-0.9 in 0.9-1.0 or 0.7-0.8. To detect which bin order achieves this best, the sampling algorithm was run multiple time, while varying the position of the 0.9-1.0 bin, while the other bins were kept constant from high to low. The average Tanimoto score per molecule was calculated and the fraction of pairs in the most sampled bin per molecule was calculated. These were used as a metric for testing how well the pairs were distributed over the Tanimoto score bins per InChiKey. Based on Supplementary Figure 10, we decided to use the bin order where we sample the bin between 0.9 and 1.0 as the third bin. This results in the average score per molecule shown in Supplementary Figure 11b.

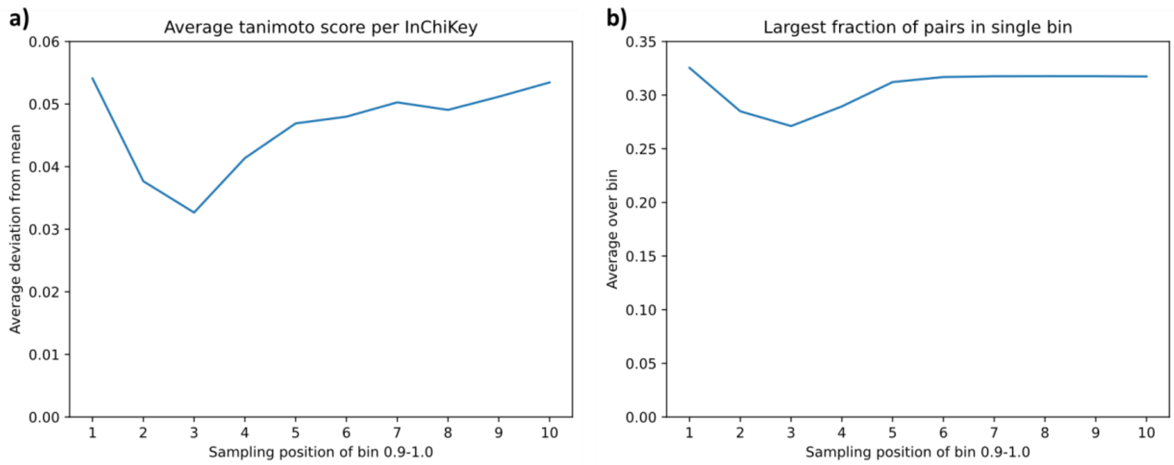

Supplementary Figure 10: Effect of sampling order. *The sampling algorithms samples pairs per bin, in later bins under sampled InChIKeys can be compensated. The sampling order has an effect on how this oversampling happens. To test the optimal sampling order, the sampling order was varied. The position of the 0.9-1.0 bin was varied, the other bins are ordered from high to low. The effect is tested on the training spectra. The raw data and notebook required to reproduce this figure is available in the Source Data file.* ***a)*** *For each molecule the average of the scores for the selected pair was calculated. The absolute difference with 0.5 (the mean) was calculated, followed by calculating the average over all molecules.* ***b)*** *For each molecule the distribution of pairs over the bins was calculated. The bin with the highest number of pairs was selected and the fraction of the total number of pairs was calculated for this molecule. A perfectly balanced score would result in 0.1 as average per bin (total/ number of bins).*

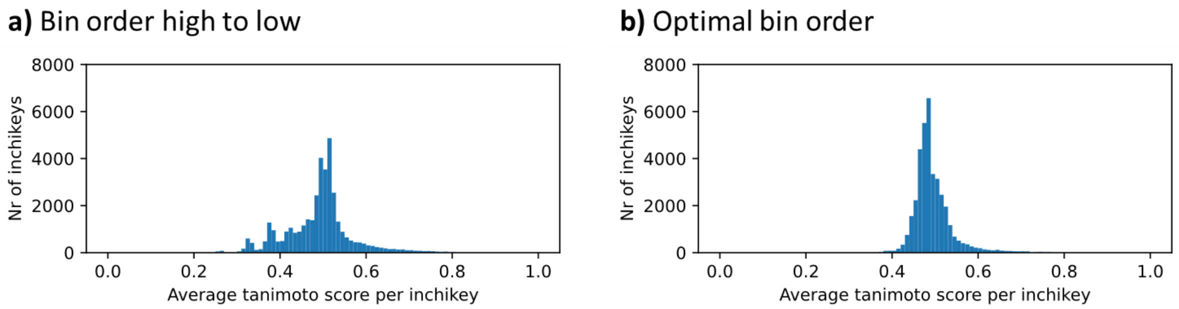

Supplementary Figure 11: Average Tanimoto score over pairs sampled per molecule for the training spectra. *The raw data and notebook required to reproduce this figure is available in the Source Data file.* ***a)*** *Sampling with bin order from high to low.* ***b)*** *Sampling with bin order [(0.8, 0.9), (0.7, 0.8), (0.9, 1.0), (0.6, 0.7), (0.5, 0.6), (0.4, 0.5), (0.3, 0.4), (0.2, 0.3), (0.1, 0.2), (-0.01, 0.1),]*

Resampling

To achieve a well-balanced Tanimoto score distribution, it is required to allow some resampling of already sampled molecule pairs. To minimize the resampling of pairs we keep track of the sampling frequency of each pair. Before selecting the least frequently sampled second molecule, we select the least frequently sampled pairs. If no limit is set, this still results in resampling some pairs 50 times, see Supplementary Figure 12a. Implementing a maximum resampling rate can reduce the frequency of resampling molecule pairs. Supplementary Figure 12 shows that setting a low maximum resampling rate results in a bad score distribution per molecule, while the number of unique pairs selected is barely affected by the resampling rate. Based on these figures, a maximum pair resampling of 20 seemed optimal. However, we recommend not to set a maximum resampling value, since the effect is minimal, and setting a too low maximum resampling setting can result in a bad balance of pairs selected per molecule.

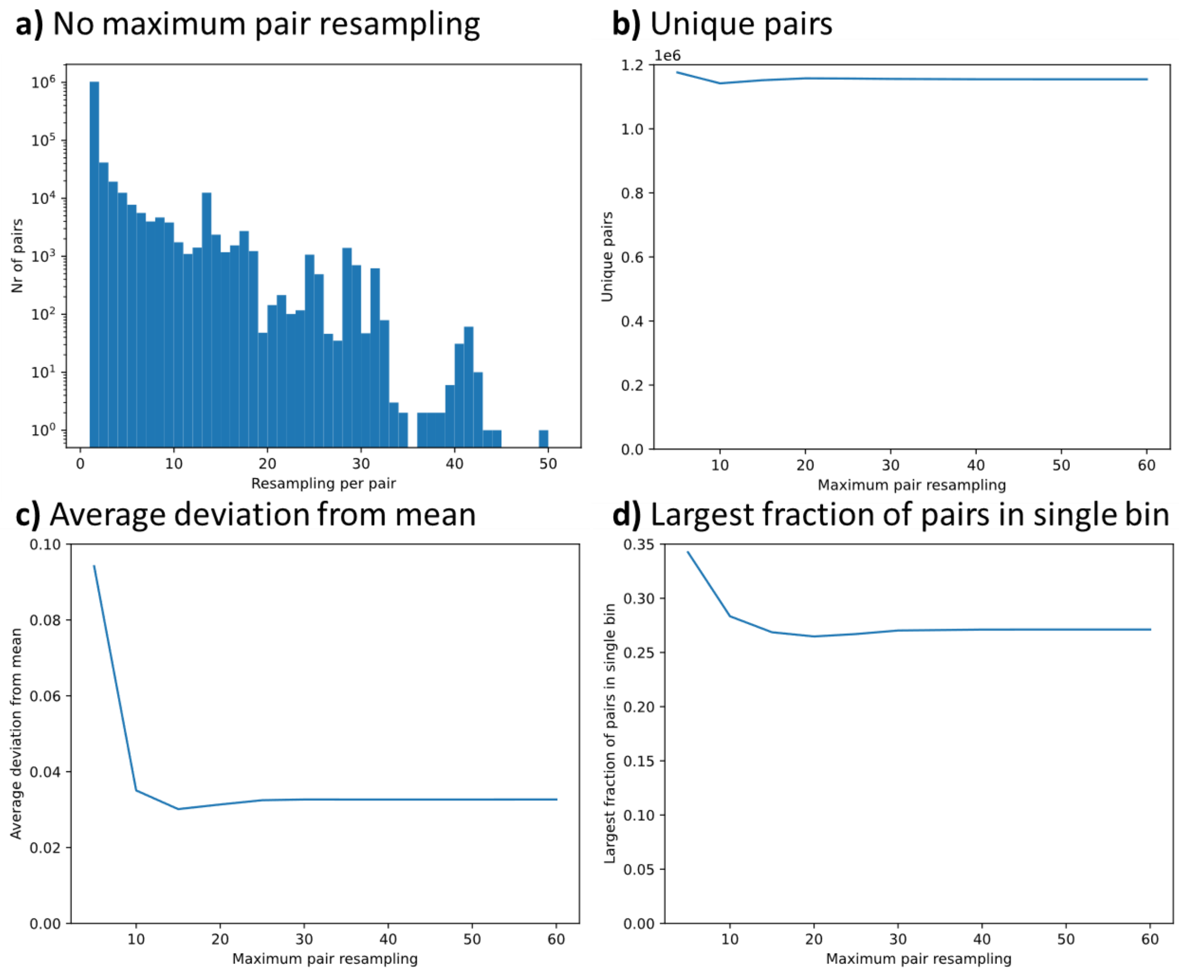

Supplementary Figure 12: Effect of setting a maximum resampling on the training spectra. *The raw data and notebook required to reproduce this figure is available in the Source Data file.* ***a)*** *The frequency of sampling each molecule pair without maximum resampling rate.* ***b)*** *The number of unique pairs sampled for different maximum pair resampling rates.* ***c)*** *For different maximum pair resampling rates the deviation from the mean Tanimoto score per molecule was calculated. For each molecule the average of the scores for the selected pair was calculated. The absolute difference with 0.5 (the mean) is calculated, followed by calculating the average over all molecules.* ***d)*** *For different maximum pair resampling rates the deviation from the mean Tanimoto score per molecule was calculated. For each molecule the distribution of pairs over the bins was calculated. The bin with the highest number of pairs was selected and the fraction of the total number of pairs was calculated for this molecule. A perfectly balanced score would result in 0.1 as average per bin (total/ number of bins).*

Number of sampled pairs

The number of pairs sampled can be varied. This can be changed by changing the average sampling count per molecule. By increasing the number of sampled pairs, the unique number of pairs increases most in the lower bins with many available pairs, while in the higher bins, with less available pairs, the number of unique pairs increases slower, since the number of pairs is mostly increased by resampling the previously selected pairs more frequently, see Supplementary Figure 13. Increasing the number of pairs sampled increases the runtime of the sampling algorithm. Sampling each molecule more than 100 times did not improve model performance (see Supplementary Figure 13b), therefore we settled for sampling each molecule 100 times on average.

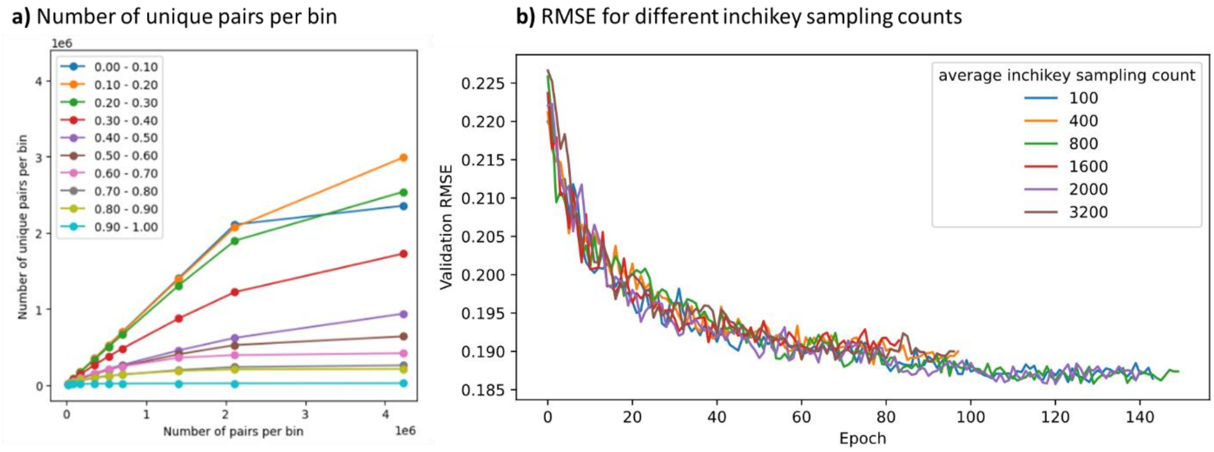

Supplementary **Figure 13: Effect of number of pairs sampled.** *The raw data and notebook required to reproduce this figure is available in the Source Data file.* **a)** Number of unique pairs per bin for different numbers of pairs sampled from the training spectra. **b)** The validation RMSE for models trained for different average InChIKey sampling counts. This shows the number of pairs sampled per InChiKey does not have a large effect on training.

Supplementary Note 3. Comparison to MS2DeepScore 0.2.0 model

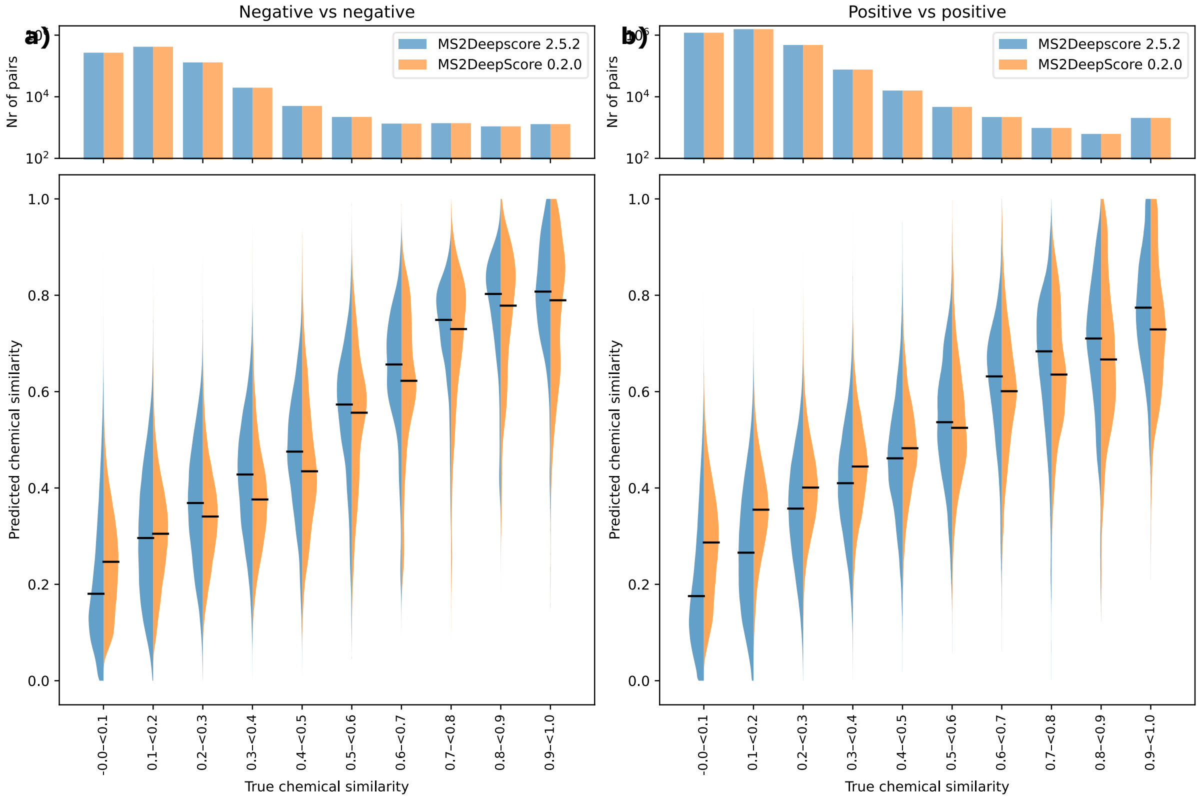

Supplementary Figure 14: Comparison between current MS2DeepScore version (2.5.2) and the original MS2DeepScore version (0.2.0). *The MS2DeepScore model 0.2.0 is the version used to train the model with the same architecture as the original MS2DeepScore paper. This model is retrained on the same training data and benchmarked on the same test set to enable comparison to the new MS2DeepScore version (2.5.4). For the 0.2.0 architecture, two models are trained, one on positive ionization mode and one on negative ionization mode spectra. Predictions are made between all test spectra, followed by taking the average per unique molecule pair. The violin plots show the kernel density estimation (KDE) of the predicted values, the black lines represent the median. The bar plot on the top shows the log-scaled count of the number of unique molecule pairs in each bin with the corresponding chemical similarity. The metric used for chemical similarity prediction is the Tanimoto score between Daylight fingerprints. The raw data and notebook required to reproduce this figure is available in the Source Data file.* ***a)*** *Predictions between pairs of negative ionization mode spectra.* ***b)*** *Predictions between pairs of positive ionization mode spectra.*

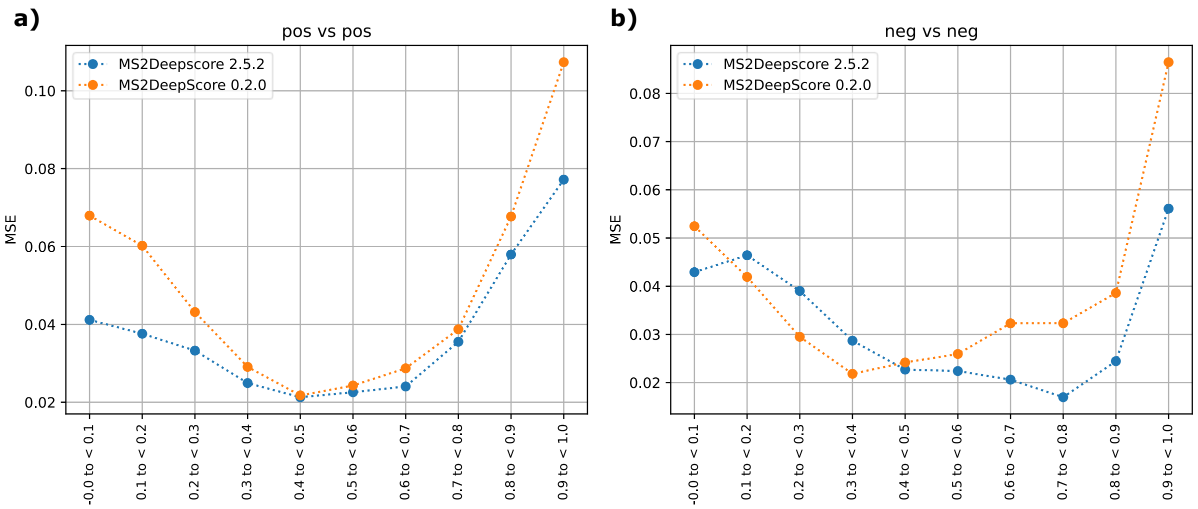

Supplementary* Figure 15: MSE per Tanimoto bin for current MS2DeepScore version (2.5.4) versus original MS2DeepScore version (0.2.0).** *Compound pairs are sampled from the test set per Tanimoto bin. The average MSE per compound pair is calculated followed by calculating the average over all compound pairs in the Tanimoto bin. The raw data and notebook required to reproduce this figure is available in the Source Data file.* ***a) *Predictions between pairs of positive ionization mode spectra.* ***b)*** *Predictions between pairs of negative ionization mode spectra.*

Supplementary Note 4: Comparison to model trained on single ionization mode

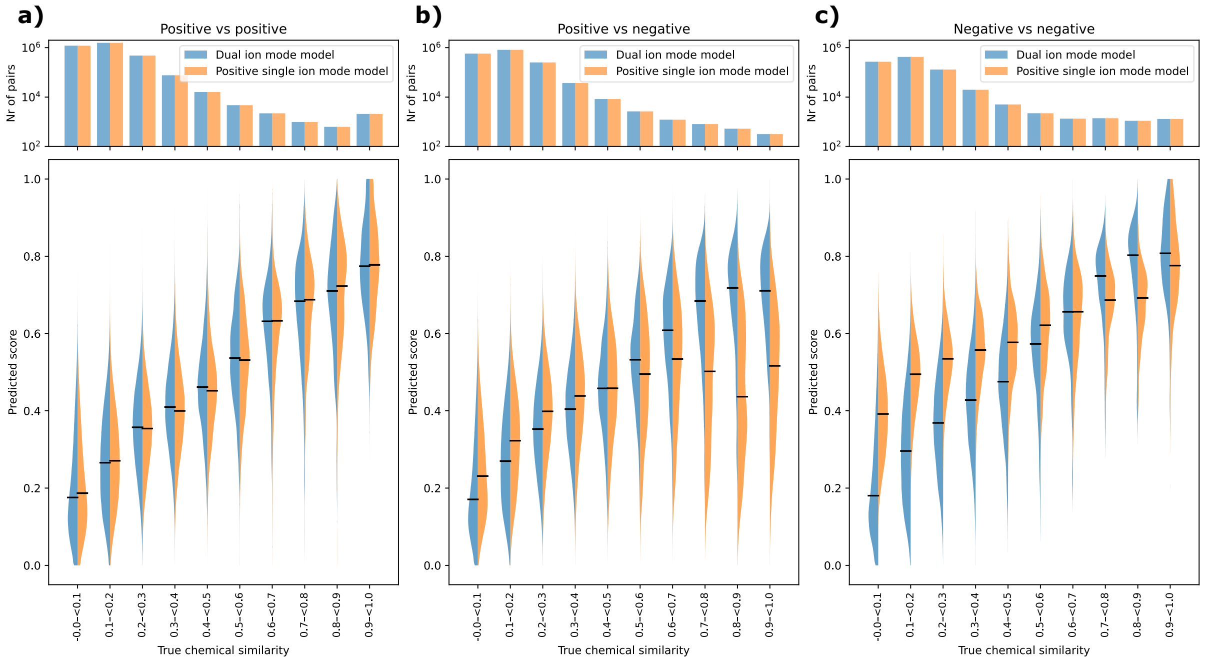

Supplementary **Figure 16*****: Comparison between cross-ionization model and model trained only on positive ionization mode spectra.*** *The model trained on only positive ionization mode spectra used the same architecture, but only trained on the positive ionization mode spectra. Predictions are made between all test spectra, followed by taking the average per unique molecule pair. The violin plots show the kernel density estimation (KDE) of the predicted values, the black lines represent the median. The bar plot on the top shows the log-scaled count of the number of unique molecule pairs in each bin with the corresponding chemical similarity. The metric used for chemical similarity prediction is the Tanimoto score between Daylight fingerprints. The raw data and notebook required to reproduce this figure is available in the Source Data file.* ***a)*** *Predictions between pairs of positive ionization mode spectra.* ***b)*** *Predictions between pairs of positive and negative ionization mode spectra.* ***c)*** *Predictions between pairs of negative ionization mode spectra.*

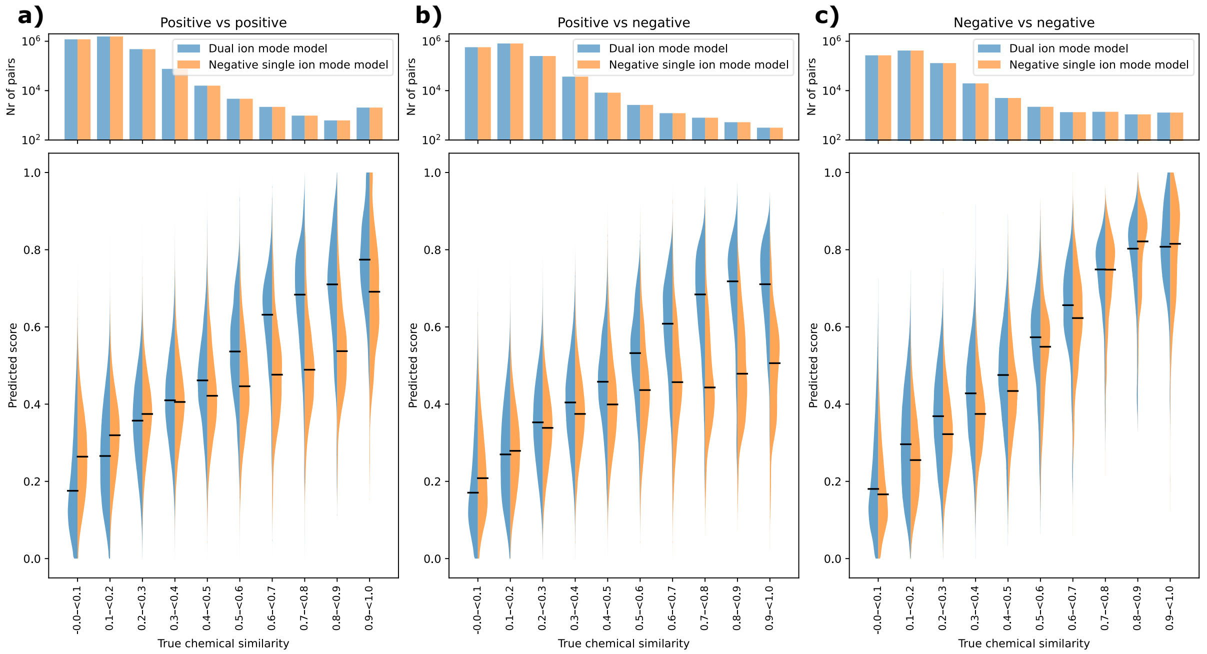

Supplementary **Figure 17: *Comparison between cross-ionization model and model trained only on negative ionization mode spectra.*** *The model trained on only negative ionization mode spectra used the same architecture, but only trained on the negative ionization mode spectra. Predictions are made between all test spectra, followed by taking the average per unique molecule pair. The violin plots show the kernel density estimation (KDE) of the predicted values, the black lines represent the median. The bar plot on the top shows the log-scaled count of the number of unique molecule pairs in each bin with the corresponding chemical similarity. The metric used for chemical similarity prediction is the Tanimoto score between Daylight fingerprints. The raw data and notebook required to reproduce this figure is available in the Source Data file.* ***a)*** *Predictions between pairs of positive ionization mode spectra.* ***b)*** *Predictions between pairs of positive and negative ionization mode spectra.* ***c)*** *Predictions between pairs of negative ionization mode spectra.*

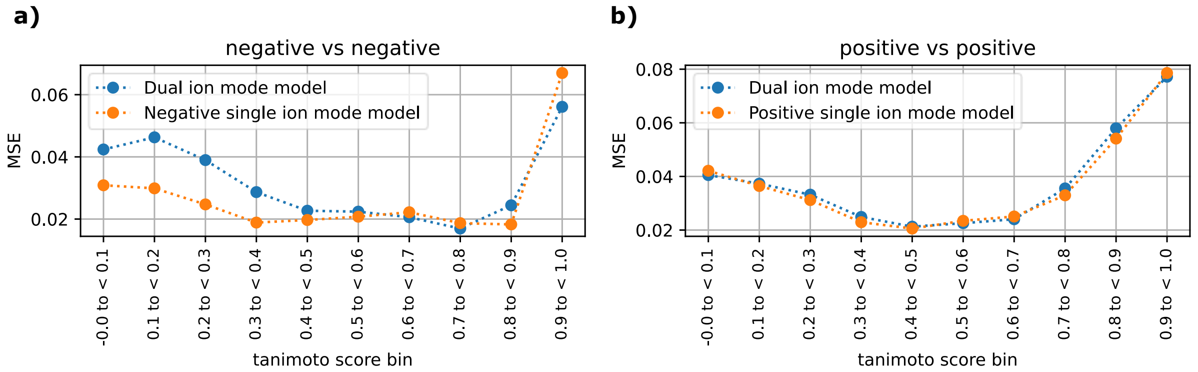

Supplementary **Figure 18: MSE per Tanimoto bin for single ionization mode model compared to dual ionization mode model.** *Compound pairs are sampled from the test set per Tanimoto bin. The average MSE per compound pair is calculated followed by calculating the average over all compound pairs in the Tanimoto bin. The raw data and notebook required to reproduce this figure is available in the Source Data file.* ***a)*** *Predictions between pairs of negative ionization mode spectra. The model trained on single ionization mode spectra performs better than the model trained on both ionization modes.* ***b)*** *Predictions between pairs of positive ionization mode spectra.*

Supplementary Note 5. Comparison to modified cosine score

The modified cosine score is not designed for predicting Tanimoto scores, so it is not expected to correlate well with the Tanimoto score. The strength of the modified cosine score is that when a high score is predicted it most of the time is a high similarity match, however, for many highly similar metabolites the modified cosine score is 0. Since the compute time of modified cosine score is significantly higher than for MS2DeepScore, it was not feasible to calculate an all vs all prediction matrix for the test set. Instead 1 spectrum per unique molecule was selected from the test set resulting in 1830 positive ionization mode spectra and 924 negative ionization mode spectra. Instead of 24911 positive mode spectra and 7142 negative mode spectra. In addition the spectra were first filtered to contain only the 100 highest intensity peaks.

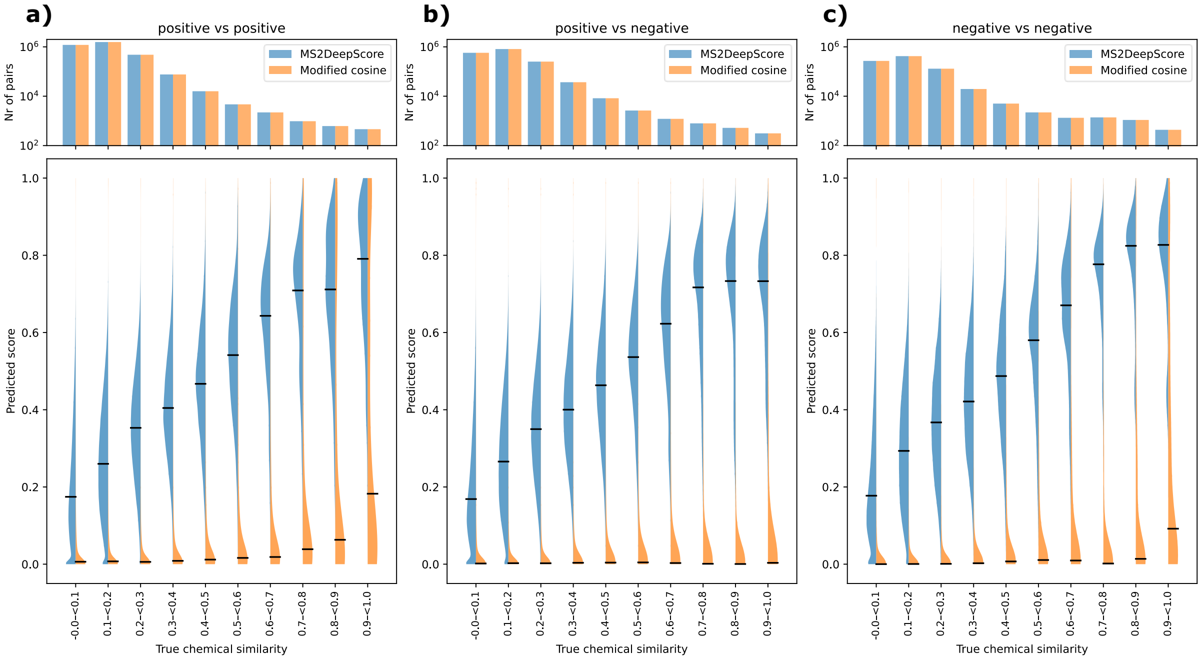

Supplementary Figure 19: Side by side violin plot of modified cosine score predictions and MS2DeepScore predictions. *One spectrum per unique molecule is randomly sampled from the test set, resulting in a subset of the test set. Modified cosine score predictions and MS2DeepScore predictions are made between all sampled spectra. The violin plots show the kernel density estimation (KDE) of the predicted values, the black line represents the median for each bin. The bar plot shows the number of unique molecule pairs for each true chemical similarity bin. The true chemical similarity used is the Tanimoto score between Daylight fingerprints. The raw data and notebook required to reproduce this figure is available in the Source Data file.* ***a)*** *Predictions between pairs of positive ionization mode spectra.* ***b)*** *Predictions between pairs positive and negative ionization mode spectra. Interestingly, MS2DeepScore models trained on only one of the ionization modes show a better than random prediction performance when predicting mass spectral similarity of spectra obtained in the other ionization mode or cross-ionization mode. This suggests that MS2DeepScore is able to detect patterns that generalize between the two ionization modes.* ***c)*** *Predictions between pairs of negative ionization mode spectra.*

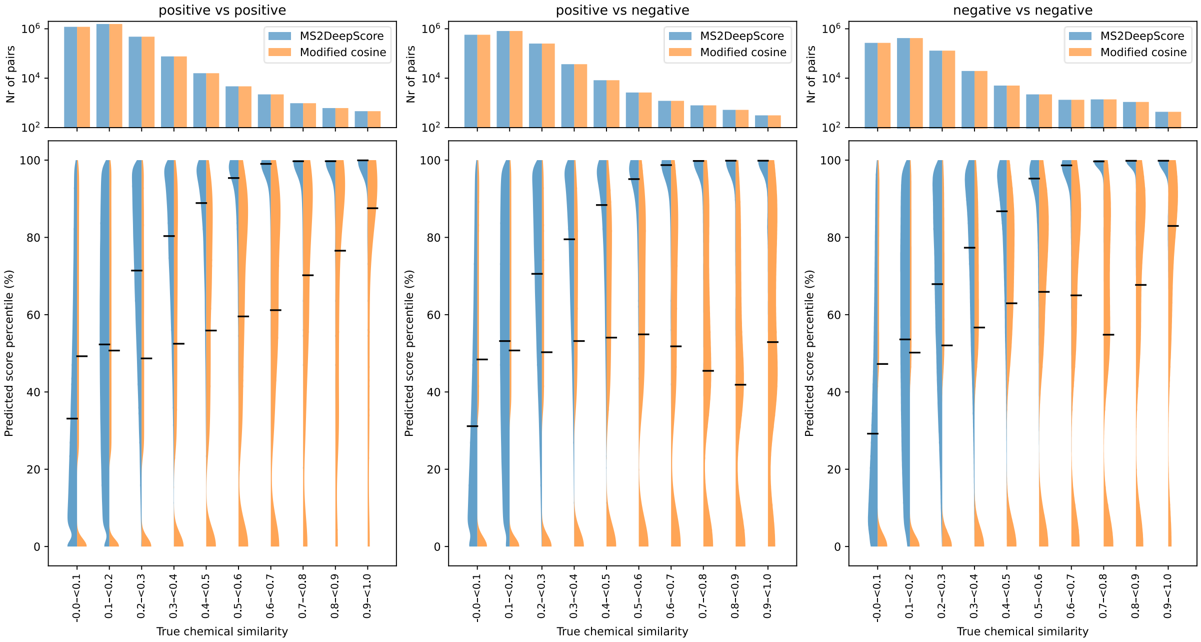

Supplementary Figure 20: Side by side violin plot of modified cosine score prediction percentiles and MS2DeepScore prediction percentiles. *One spectrum per unique molecule is randomly sampled from the test set, resulting in a subset of the test set. Modified cosine score predictions and MS2DeepScore predictions are made between all sampled spectra. The predictions are converted to predicted percentiles, by ranking the scores over all predictions made for the used small test set of each ion mode, this enables a better comparison, since it converts the predictions to a more comparable scale. The violin plots show the kernel density estimation (KDE) of the predicted values, the black line represents the median for each bin. The bar plot shows the number of unique molecule pairs for each true chemical similarity bin. The true chemical similarity used is the Tanimoto score between Daylight fingerprints. The raw data and notebook required to reproduce this figure is available in the Source Data file.* ***a)*** *Predictions between pairs of positive ionization mode spectra.* ***b)*** *Predictions between pairs positive and negative ionization mode spectra. Interestingly, MS2DeepScore models trained on only one of the ionization modes show a better than random prediction performance when predicting mass spectral similarity of spectra obtained in the other ionization mode or cross-ionization mode. This suggests that MS2DeepScore is able to detect patterns that generalize between the two ionization modes.* ***c)*** *Predictions between pairs of negative ionization mode spectra.*

Supplementary Note 6. Embedding Evaluator

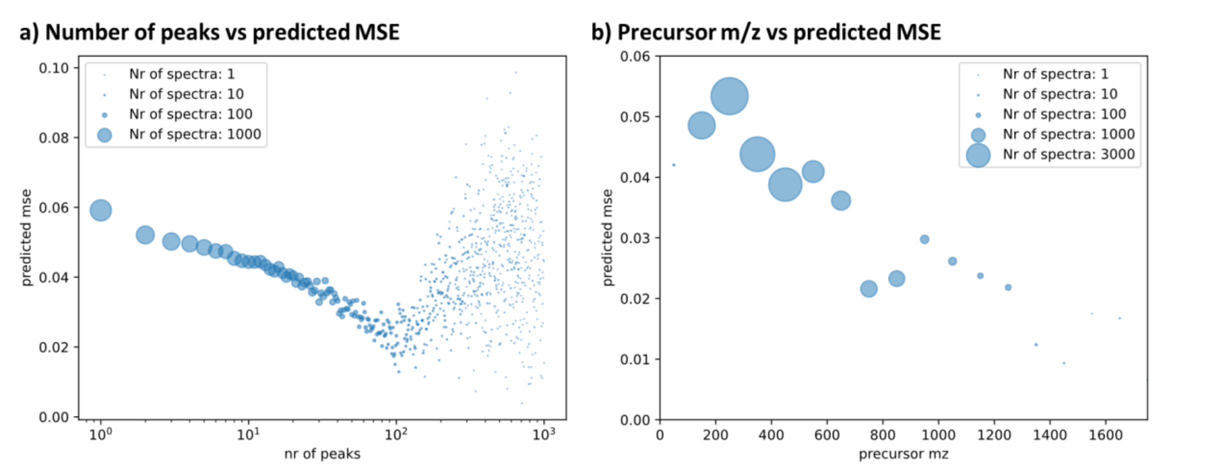

***Supplementary* Figure 21: Relationship between predicted MSE and number of peaks and precursor *m/z*.** For all test spectra the MSE is predicted using our Embedding Evaluator model. *The raw data and notebook required to reproduce this figure is available in the Source Data file.* **a)** The number of peaks is plotted against the predicted MSE. The average of all spectra with a specific number of peaks is plotted. The size of the dot shows the number of spectra in the test set that have this number of peaks. **b)** The precursor *m/z* is plotted against the predicted MSE. The average of all spectra in bins of 100 Da is plotted. The size of the dot shows the number of spectra in the test set that fall in this precursor *m/z* bin.

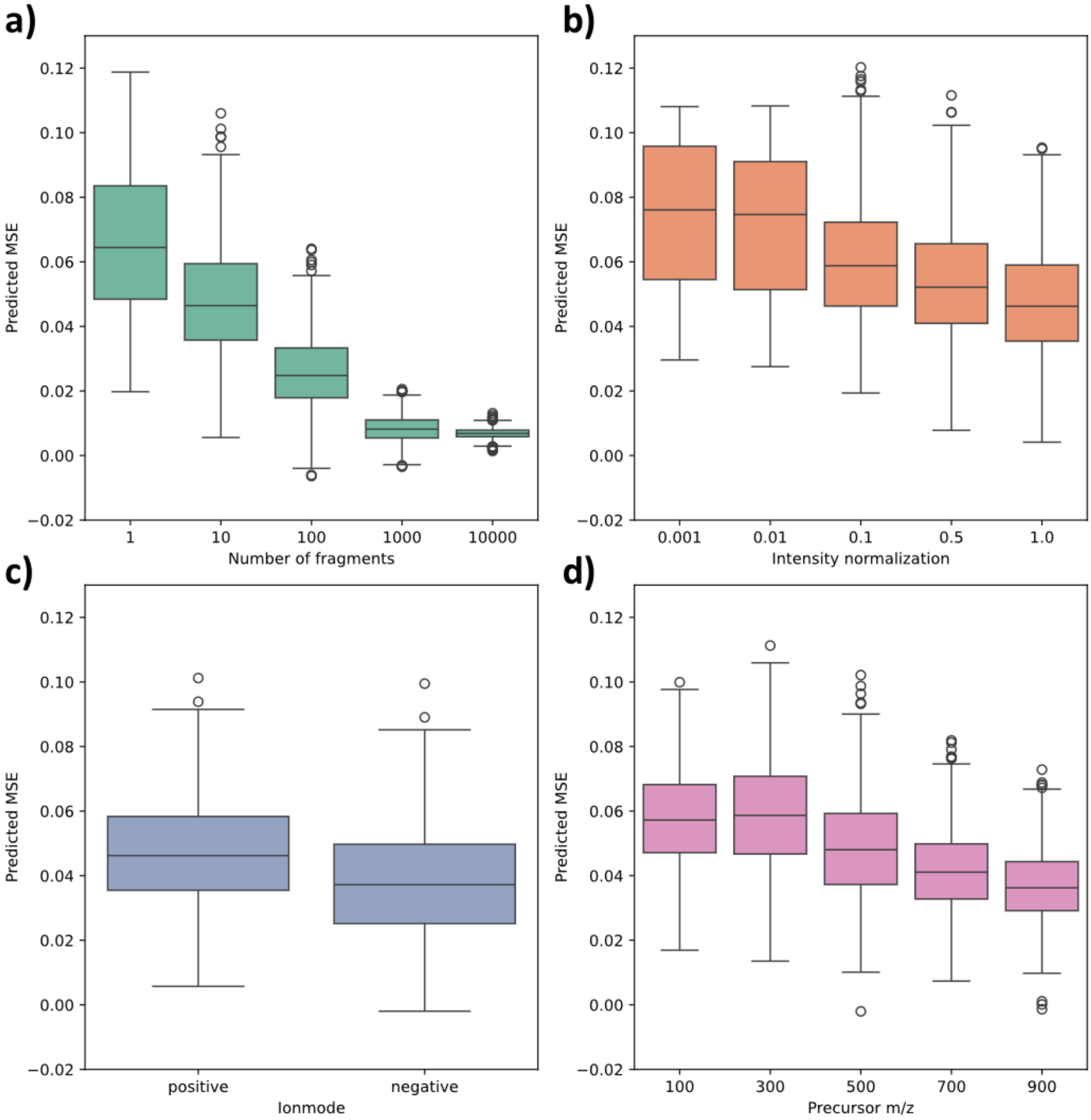

Supplementary Figure 22: Predicted MSE for randomly generated spectra. *A 1000 spectra are randomly generated for each test by creating random fragments, with random intensities and random precursor m/z. The default number of fragments was 10, the default intensity was between 0 and 1, the default m/z values of the fragments were between 10 and 1000 Da, the default ionization mode was positive and a random precursor m/z was selected between 0 and 1000 Da. The raw data and notebook required to reproduce this figure is available in the Source Data file.* ***a)*** *The number of fragments per randomly generated spectrum is varied.* ***b)*** *The maximum randomly generated intensity of the peaks is varied, e.g. 0.01 means random intensities are generated between 0 and 0.01.* ***c)*** *The ionization mode for all spectra is set to positive or negative.* ***d)*** *The precursor m/z is fixed to a specific value.*

Supplementary Note 7. Detailed analysis of MS2DeepScore model

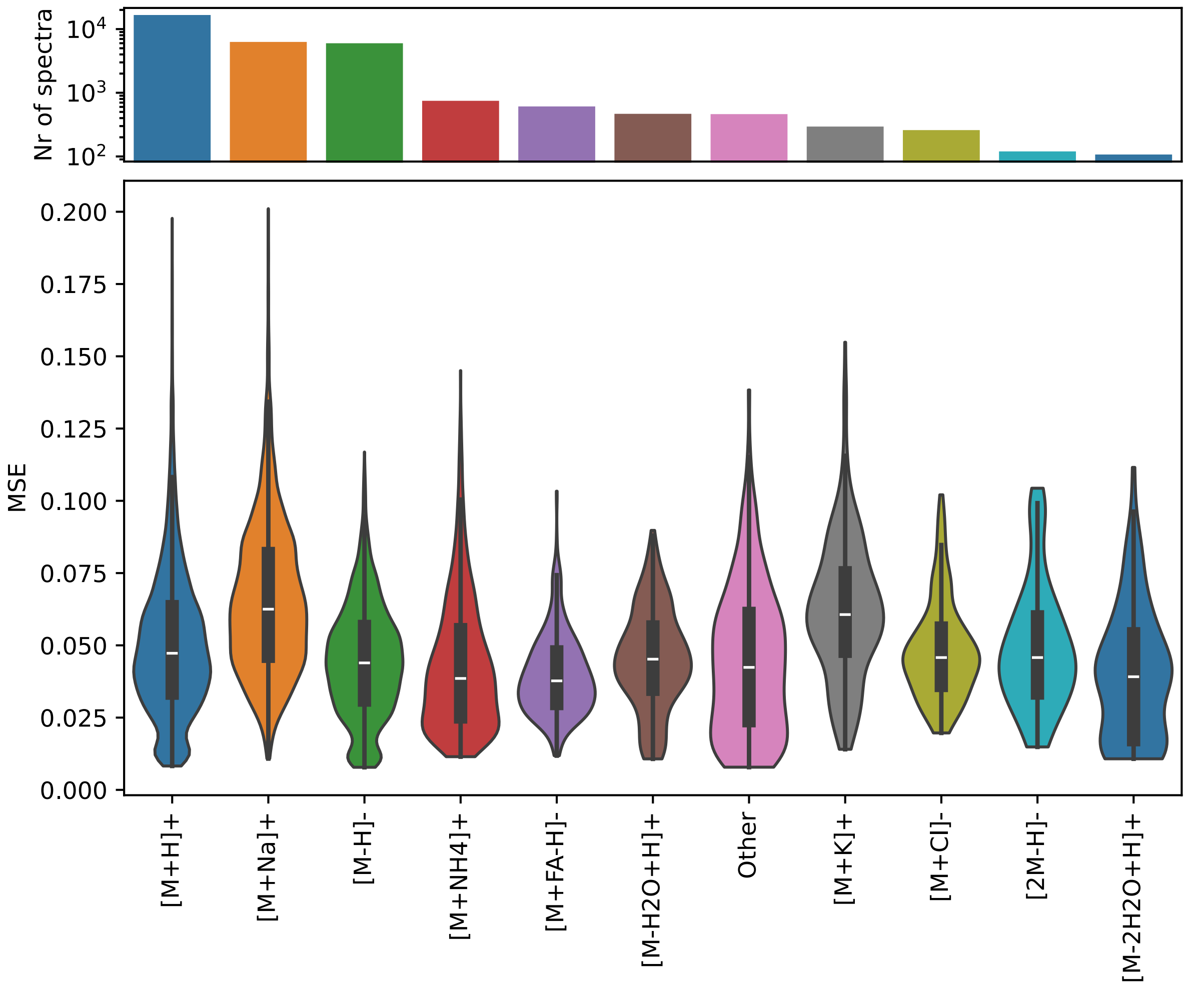

Supplementary Figure 23: MSE per adduct type. *For the test set the MSE per spectrum was calculated. Here the distribution of MSE per adduct type is given. Any adduct with less than 100 spectra is combined in the "other" boxplot. The raw data and notebook required to reproduce this figure is available in the Source Data file.*

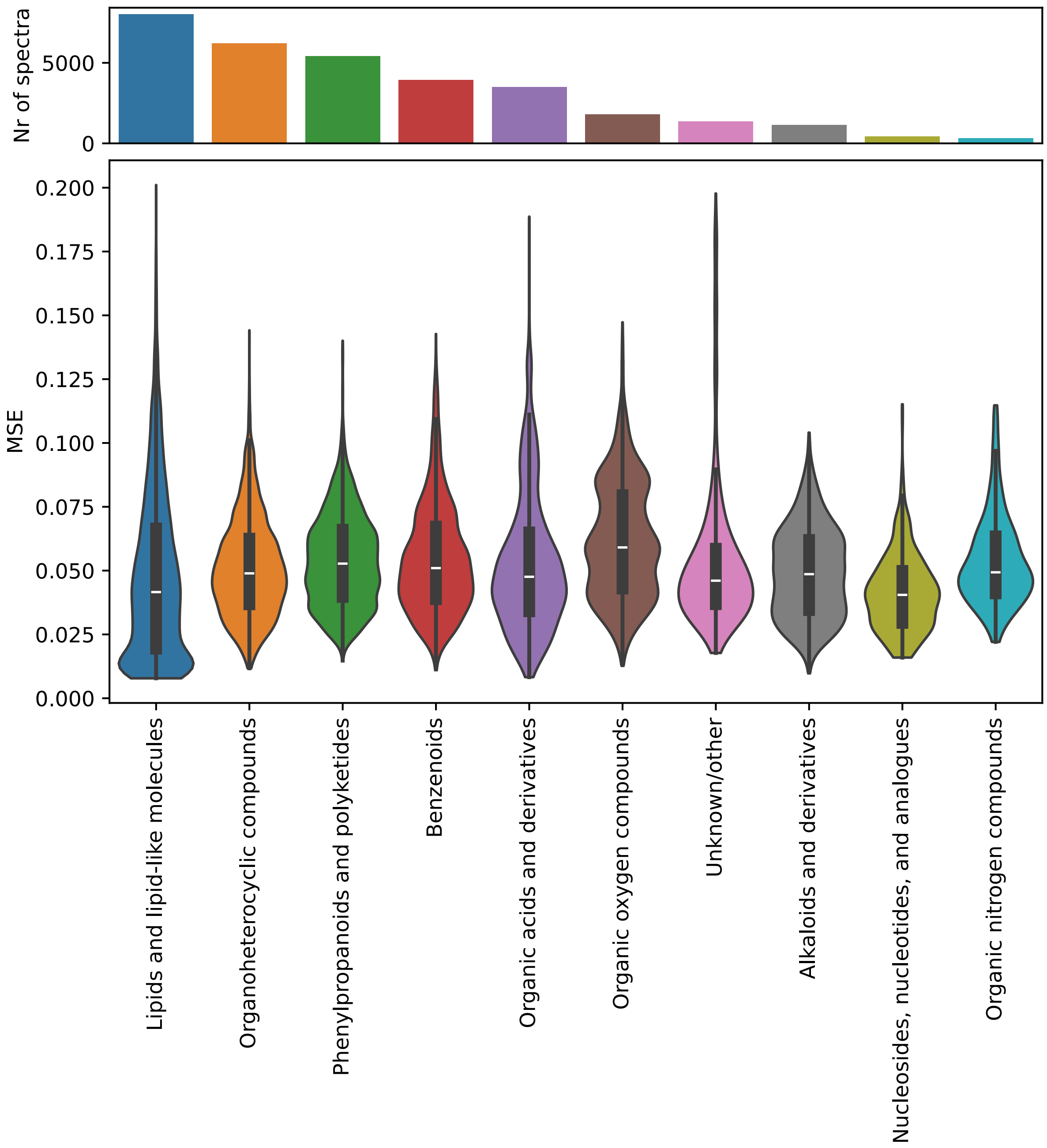

Supplementary Figure 24: MSE per chemical class. *For the test set the MSE per spectrum was calculated. Here the distribution of MSE per chemical class is given. The chemical class is determined by using ClassyFire[3], both molecules for which no ClassyFire annotation was available and chemical classes with less than 300 spectra are combined in the boxplot Unknown/other. The raw data and notebook required to reproduce this figure is available in the Source Data file.*

Supplementary Note 8. Human blood plasma case study

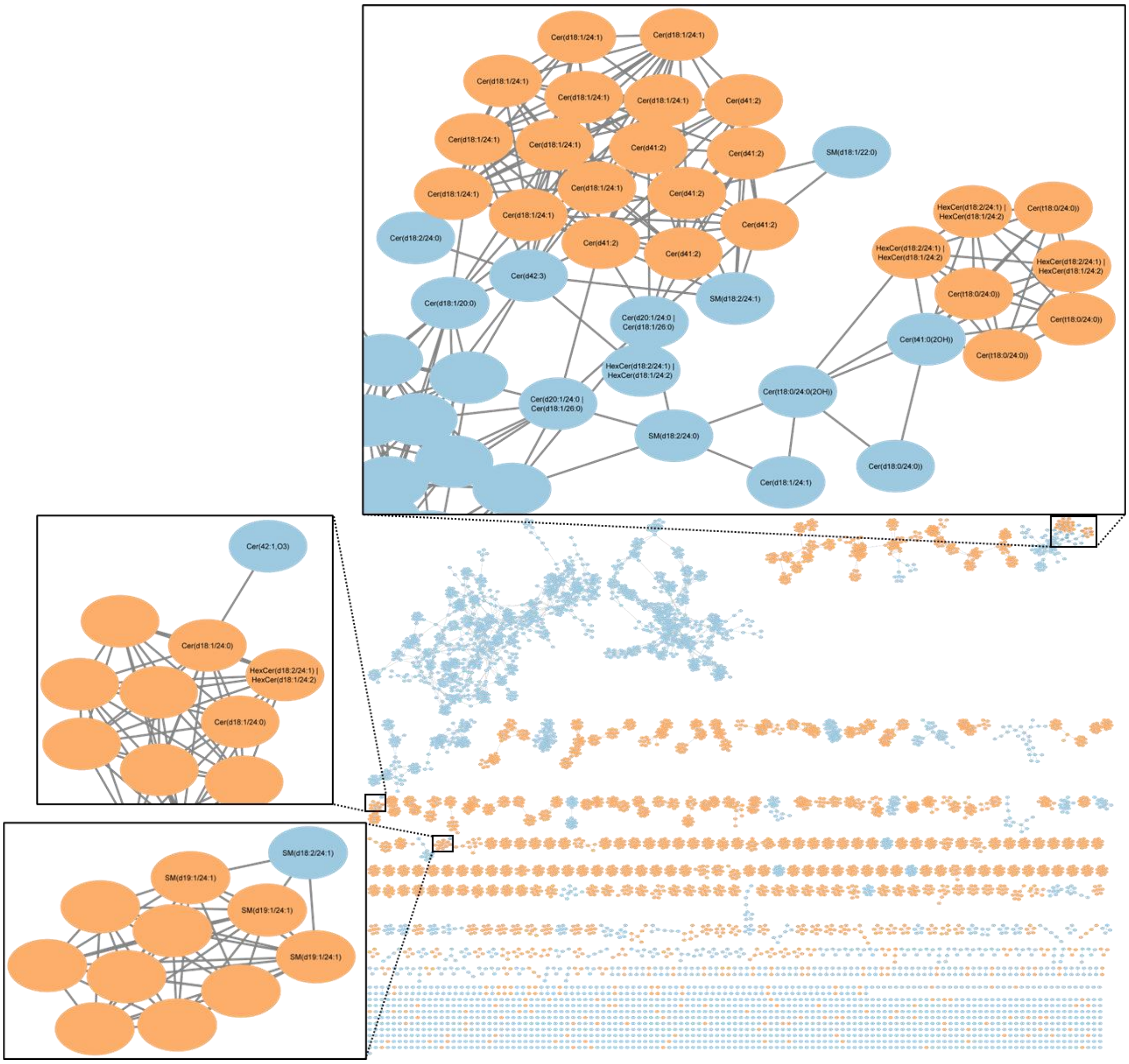

Supplementary Figure 25: Molecular network created with MS2DeepScore cross-ionization-mode model on human blood plasma case study. *By predicting chemical similarity between both the positive and negative ionization mode spectra, spectra of both ionization modes can be visualized together. An edge is created for an MS2DeepScore larger than 0.85. We highlight a few examples where MS2DeepScore was able to predict close chemical similarity between positive and negative ionization modes. The annotations were added by manual annotations. The orange nodes correspond to negative ionization mode spectra and blue nodes correspond to positive ionization mode spectra. The raw data and notebook required to reproduce this figure is available in the Source Data file.*

Supplementary Note 9. Spectrum comparison plots

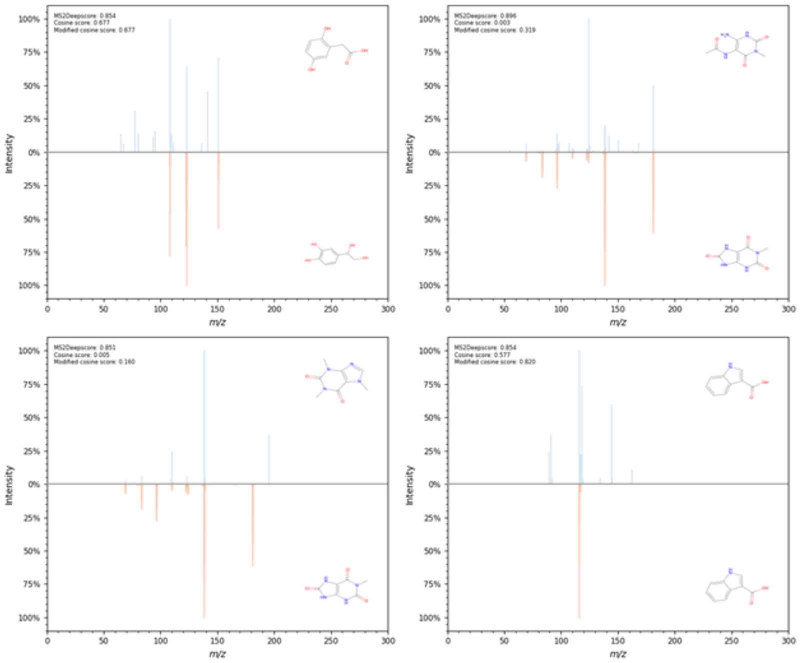

Supplementary Figure 26: Spectrum comparisons of positive and negative ionization mode spectra for which MS2DeepScore predicts high chemical similarity. *The spectra are from the urine case study. Positive ionization mode spectra are blue and negative ionization mode spectra are orange. The raw data and notebook required to reproduce this figure is available in the Source Data file.*

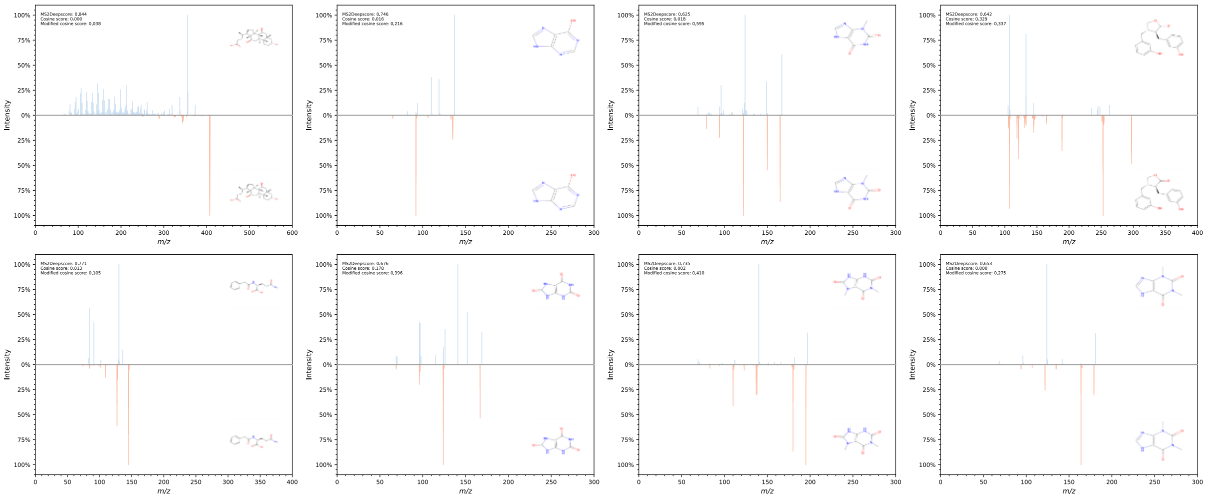

Supplementary Figure 27: Spectrum comparisons of positive and negative ionization mode spectra from the urine case study. *Structures are putatively annotated through MS2Query predictions and all have a MS2Query score of at least 0.8 and a mass difference < 0.1 for the precursor m/z. Spectrum pairs are selected for which a positive and negative ionization mode spectrum was detected, with identical annotation. Positive ionization mode spectra are blue and negative ionization mode spectra are orange. The cosine score, the modified cosine score and the MS2DeepScore prediction are given between each spectrum pair. The raw data and notebook required to reproduce this figure is available in the Source Data file.*

Supplementary Note 10: Benchmarking specific use cases

Additional benchmarking tests are performed to illustrate accuracy on downstream tasks like molecular networking and analogue searching. To this end, the test set was used as described in the methods section, "Input data filtering and splitting". This is a random subset of the annotated public libraries. For each query spectrum, the best possible analogue search hit is selected from the rest of the test set, including both the positive and negative spectra. This selection is based on the highest Tanimoto score, excluding any exact matches. After selecting the best possible analogue, the MS2DeepScore model is used to rank all mass spectra in the test set for each query spectrum. The percentage that the best possible analogue is ranked in the top 1, top 3 or top 10 is given. The analysis is repeated for searching in each ion mode or searching in both ionization modes. The result is weighted to ensure each unique InChIKey counts equally. Supplementary Table 2 shows that searching in both ionization modes does not impact how often the best hit is found in the top 1, but results in an increased frequency of selecting the best possible match within the top 10.

**Query ion mode**	**Library ion mode**	**Best analogue in top-1**	**Best analogue in top-3**	**Best analogue in top-10**
Negative	Negative	1.1%	4.0%	8.4%
Negative	Negative + positive	1.1%	3.9%	9.6%
Negative	Positive	2.0%	3.4%	6.0%
Positive	Positive	3.9%	8.0%	12.9%
Positive	Positive + negative	3.9%	8.3%	13.4%
Positive	Negative	1.2%	1.8%	3.2%

Supplementary Table 2: Percentage of best possible analogue in top k. The best possible analogue is selected from all available InChIKeys, in both positive and negative ionization modes.

To qualitatively illustrate the accuracy of the top predictions, additional benchmarking is performed. From the test set, a single spectrum is selected at random for each unique molecule. From this subset, 924 spectra are sampled from both the positive and negative ion mode spectra to have an equal number of mass spectra for each ion mode. Predictions are made between all the selected mass spectra using the new MS2DeepScore model, both within and across ionization modes. The predictions are ranked, and the top k highest predictions are selected that are above a minimum threshold. To illustrate a molecular networking scenario, we use the top 10 hits, and to illustrate an analogue searching scenario, we use the top 1 hit. For these highest-ranked predictions, the error is computed by subtracting the real Tanimoto score from the predicted Tanimoto score. From all 1848 mass spectra the top k are selected that have a score higher than the minimum threshold. The top predictions are separated into four cases, pos-pos, neg-neg, pos-neg, and neg-pos.

Supplementary Figure 28 illustrates a case like analogue searching where the top 10 hits are selected, that have a minimum threshold of 0.85. These are the same settings used as in the case studies illustrated in Figure 3. The test set used here is a more challenging case than the case studies, since the molecules in this test set are randomly selected from all available training data and are therefore expected to be much more diverse than a normal sample, therefore having less available high chemical similarity pairs within the dataset than a normal dataset. Therefore, the case studies represented in Figure 3 give a more realistic overview on how the tool is expected to perform on real data to create a molecular network.

In Supplementary Figure 28 and 29 we use a minimum threshold of 0.85. Using such a threshold is recommended, since not using a threshold results in too many false positives. This is illustrated in Supplementary Figure 30 and 31, here no threshold is used, which results in a lot of false positives.

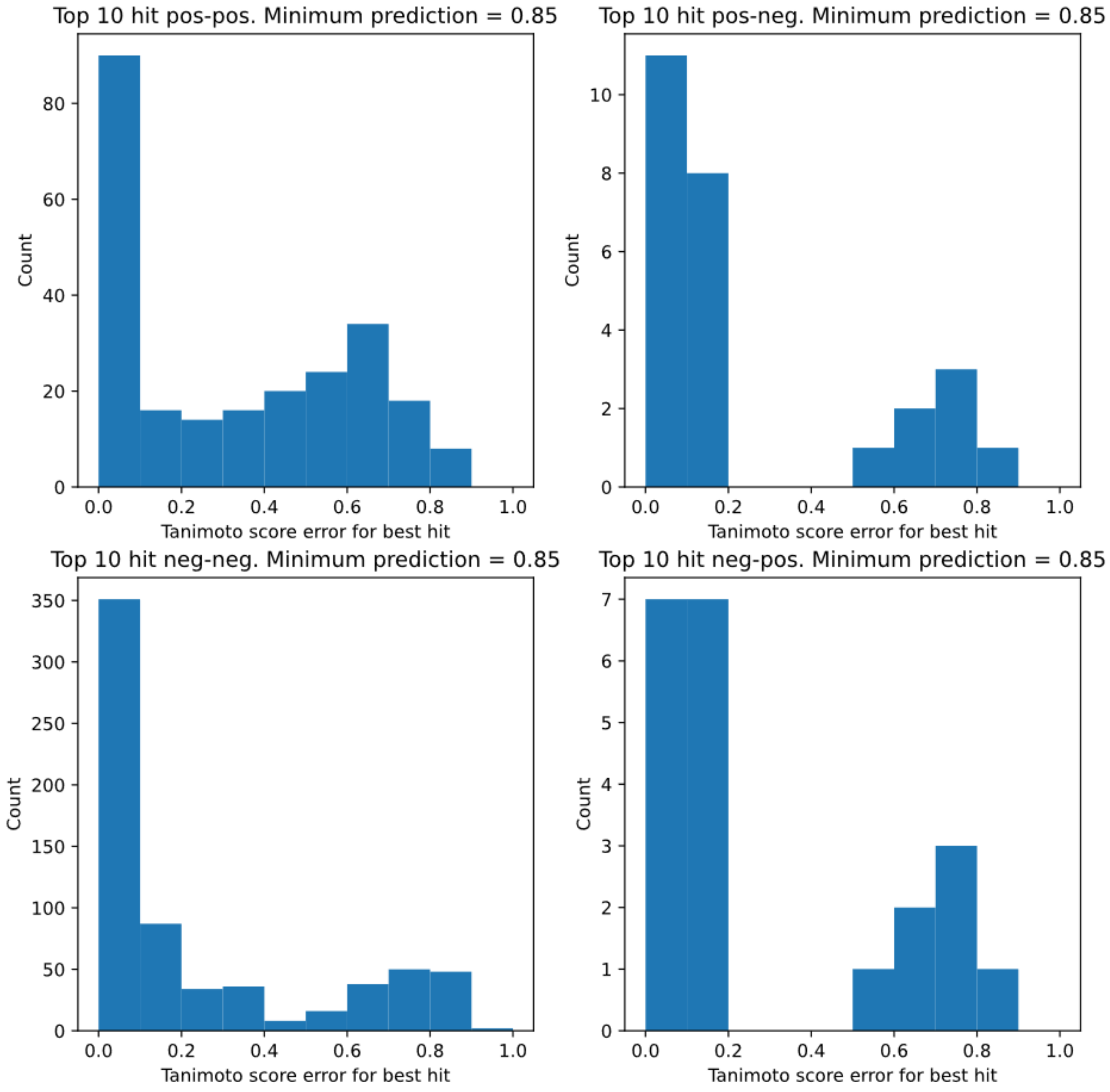

Supplementary Figure 28: Error for top-10 highest predictions above 0.85. *For each mass spectrum, the top 10 highest predictions are selected that have a predicted score of at least 0.85. The error is the difference between the predicted score and the real Tanimoto score. The raw data and notebook required to reproduce this figure is available in the Source Data file.*

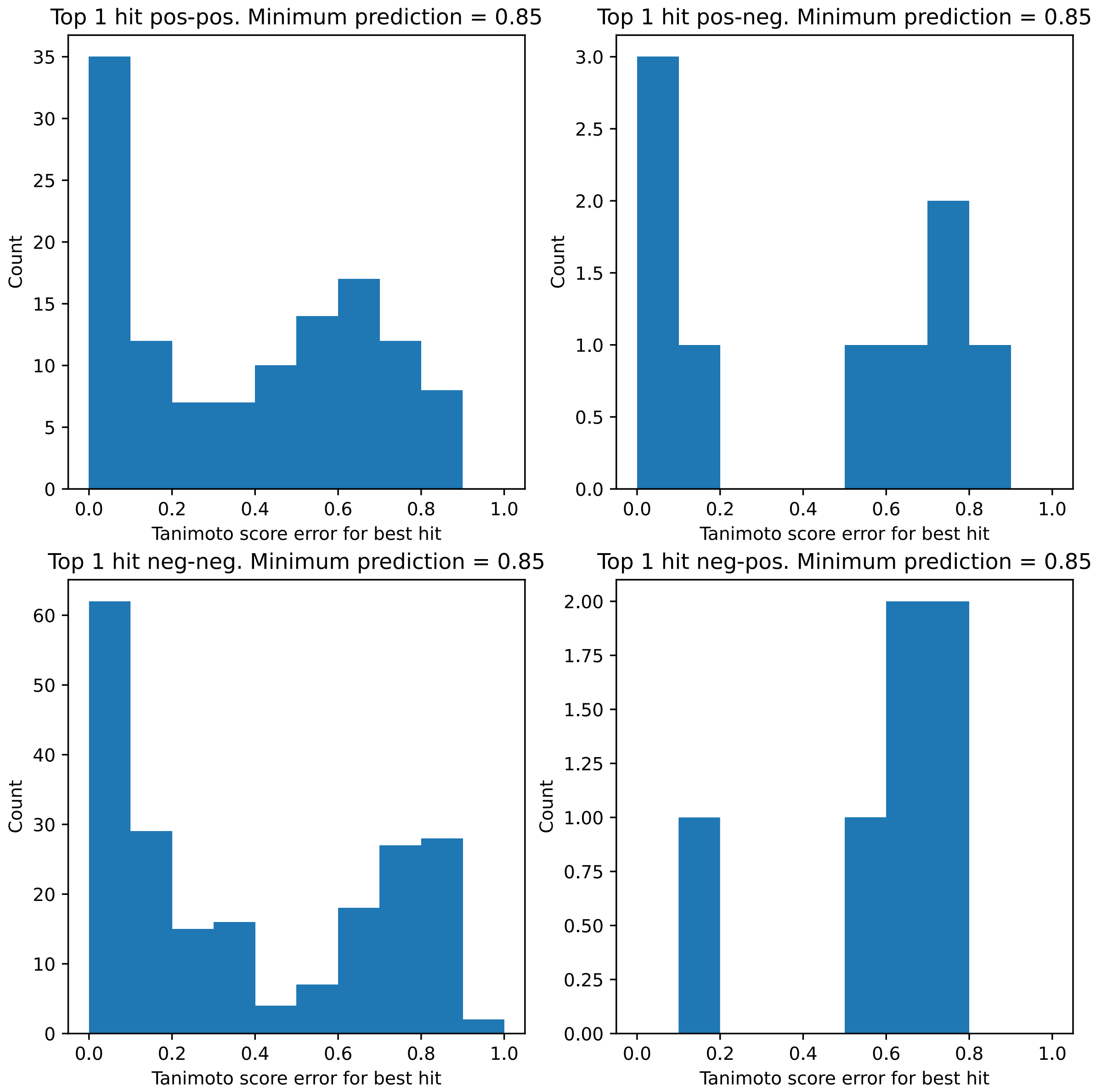

Supplementary Figure 29: Error for top-1 highest predictions above 0.85. *For each mass spectrum, the top 1 highest predictions are selected that have a score of at least 0.85. The error is the difference between the predicted score and the real Tanimoto score. The raw data and notebook required to reproduce this figure is available in the Source Data file.*

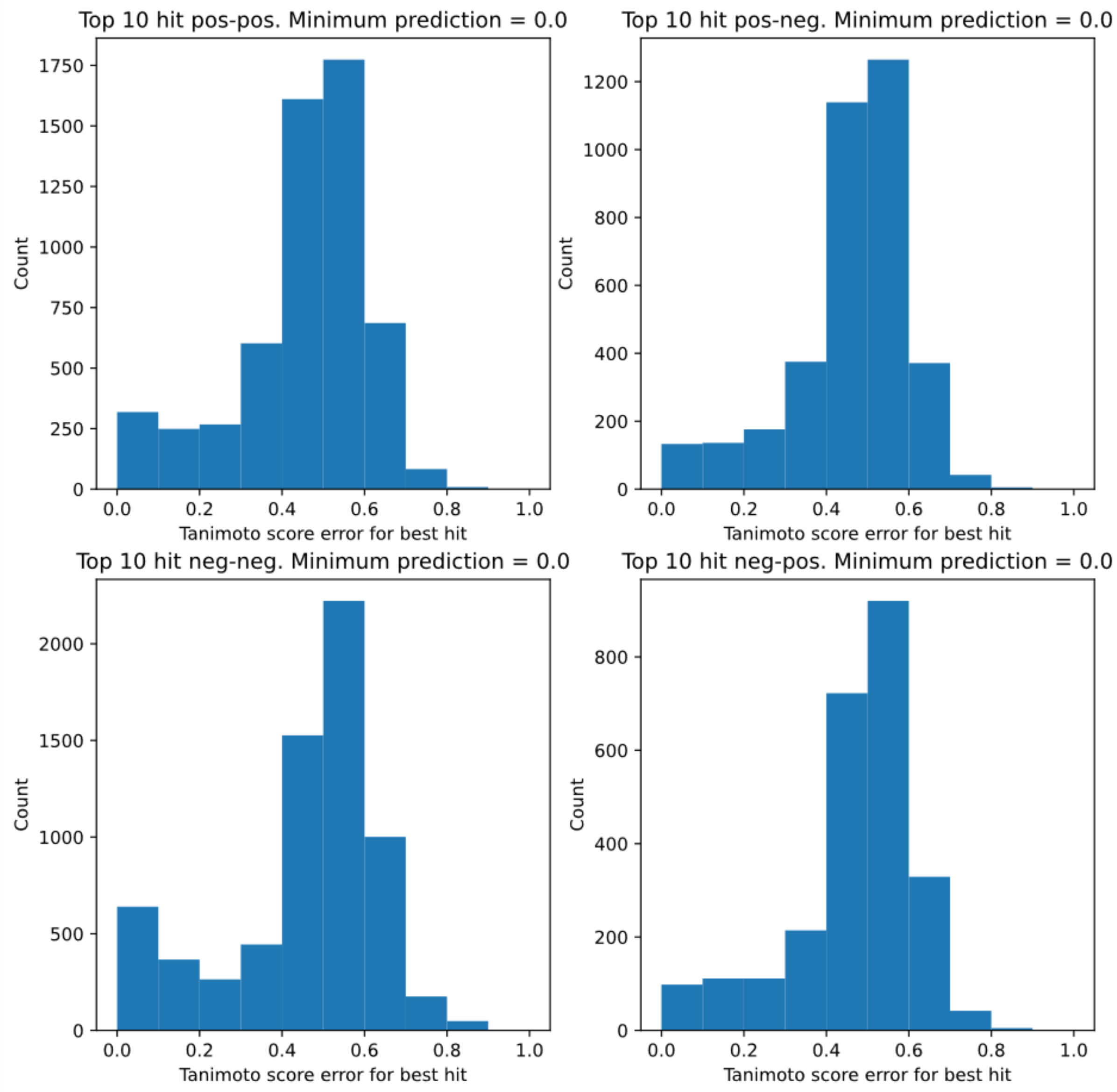

Supplementary Figure 30: Error for top-10 highest predictions, without filtering on a minimal score. *For each mass spectrum, the top 10 highest predictions are selected, without filtering on a minimum score. This is not recommended, since it results in many false positives. It is recommended to set a high minimal chemical similarity score. The error is the difference between the predicted score and the real Tanimoto score. The raw data and notebook required to reproduce this figure is available in the Source Data file.*

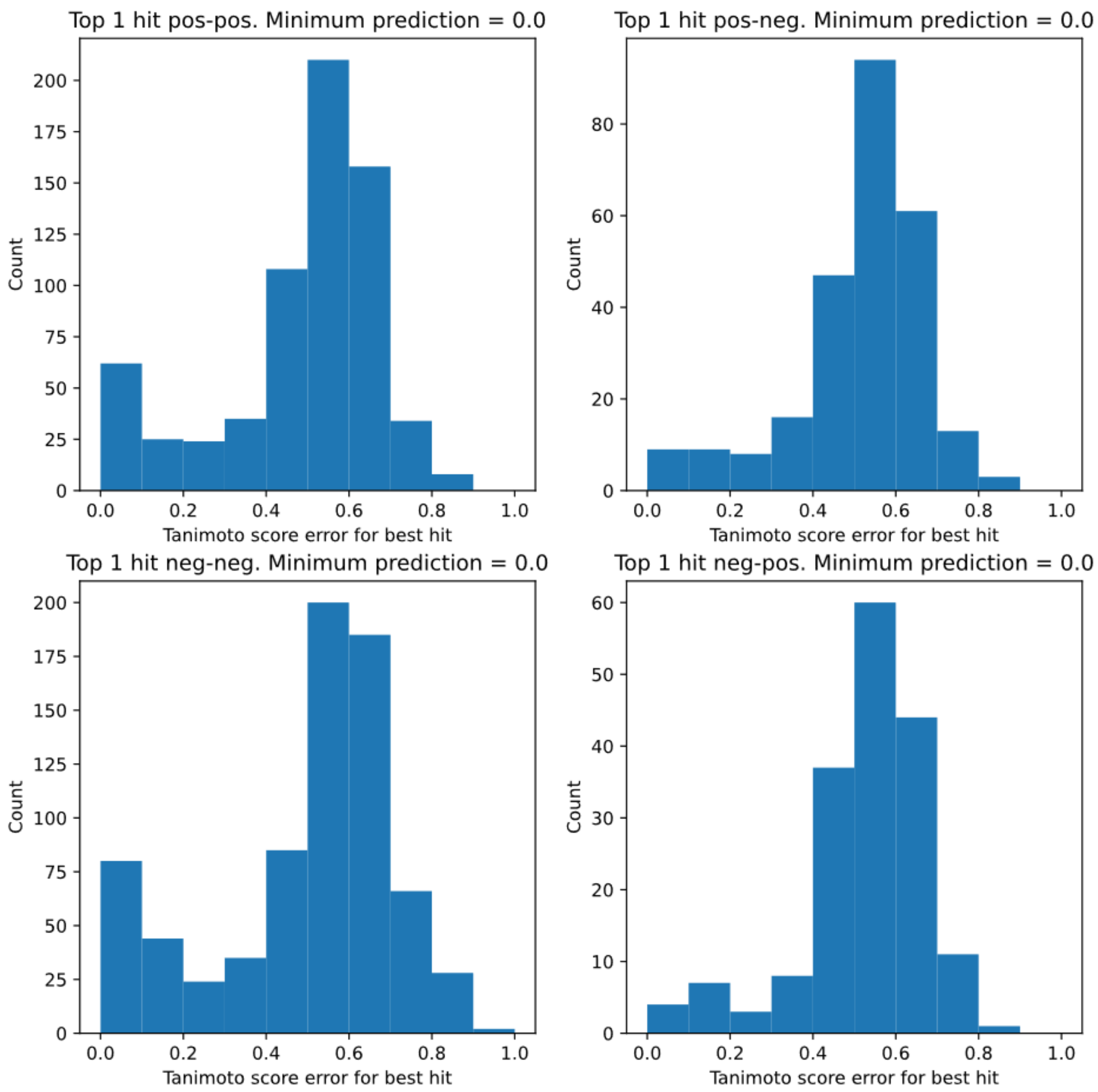

Supplementary Figure 31: Error for top-1 highest predictions, without filtering on a minimal score. *For each mass spectrum, the top 1 highest predictions are selected, without filtering on a minimum score. This is not recommended, since it results in many false positives. It is recommended to set a high minimal chemical similarity score. The error is the difference between the predicted score and the real Tanimoto score. The raw data and notebook required to reproduce this figure is available in the Source Data file.*

Supplementary Methods

Urine fractions generation

A bank of 90 human urine fractions was created as described in Albreht et al.[4] from pooled urine sample collected from healthy individuals (10-fold pre-concentrated to 1.5 L) using a semi-preparative system consisting of two analytical HPLC pumps (PerkinElmer 200 series (PerkinElmer, Waltham, MA, USA) and Agilent 1100 (Agilent, Santa Clara, CA, USA) HPLC quaternary pump), which were run in parallel, and a Waters Fraction Collector III (Waters, Milford, MA, USA). RP-LC fractionation was achieved using a preparative Capital HPLC LTD ODS-H-OL5 column (150 x 20 mm I.D., 5 µm; Capital HPLC LTD, Broxburn, UK) at room temperature with a flow rate of 20 mL/min delivered by two HPLC pumps (each performing at 10 mL/min) and loading 20 mL of pooled urine onto the column in each run. Mobile phase A was 0.1% of formic acid in water:ACN (98:2), and solvent B was 0.1% of formic acid in ACN. The sample was introduced for 1 min at the beginning of each fractionation run applying the following gradient immediately after: 0% B (1 – 2 min), 0 – 55% B (2 – 12 min), 55 – 100% B (12 – 12.5 min), 100% B (12.5 – 17 min), and 0% B (17 – 24 min). Fractions were collected from 1 – 16 min in 10 s time intervals which yielding 90 consecutive RP fractions (*ca*. 170 mL of each pooled individual fraction). The collected RP fractions were stored at −80 °C in individual 250 mL HDPE rectangular containers until use.

Urine fractions profiling

RP urine profiling was completed using a Waters Acquity UPLC system coupled to a Waters Xevo G2 QToF mass spectrometer (Waters Corp., Milford, MA, USA). LC separation was conducted on a 2.1 × 150 mm HSS T3 column (Waters Corp., Milford, MA, USA) maintained at 45 °C. Mobile phase flow rate was of 0.6 mL/min. A gradient was applied consisting of 0.1% formic acid in water (A) and 0.1% formic acid in ACN (B). Initial conditions of 99% A were held at isocratic conditions for 0.1 min followed by a linear gradient elution, 99% A to 45% A, in 9.9 min, and a final rapid gradient phase, 45% A to 0% A in 0.7 min, prior to returning to initial conditions. Injection volume was of 2µL.

Each RP fraction was prepared for the UHPLC-RP analysis from 150 µL aliquot of each fraction diluted with 75 µL of ultrapure water and 75 µL of RPC-specific internal standards (IS) solution[5]. The samples were mixed at 850 rpm for one minute at 4°C and centrifuged for 10 mins at 3486×g at 4°C. The supernatants were aspirated and dispensed into LC-MS vials for the analysis. Urine sample was injected (2µL) in the chromatographic system using full loop injection mode.

Mass spectrometry parameters were the following: capillary voltage was set at 1.5 and 1.0kV for positive and negative ionization modes, respectively. Cone voltage (20V), source offset (80V), StepWave 2 offset (10V) and gas flows of 150 L/hr for cone gas and 1000L/hr for desolvation gas were consistent for both polarities. The mass spectrometer was operating in Fast DDA mode. The intensity threshold of precursor ion was set to 100 K to trigger MS/MS fragmentation that was performed in centroid mode with a scan range of 50-1200 *m/z* and a scan time of 0.25 s. MS/MS was switched back to MS survey function after 2 s of acquisition. Deisotoped peak selection option was enabled. The collision energy was set to the ramp of 15–30 eV and 30-60 eV for MS/MS acquisition of low and high mass ions, respectively. Ten iterative DDA acquisitions were performed using DDA auto exclude program, which allows ions selected as precursors in previous injections to be removed from the list in the following injections.

Sample preparations, LC conditions and gradient elution program Human blood plasma

NIST 1950 human plasma sample was thawed at 4 °C for 2 h, from which a 50 µL aliquot was taken and diluted with LC-MS grade water (1:1 v/v) and four parts of isopropanol (IPA) containing a mixture of lipid reference standards to one part of diluted sample for protein precipitation. The sample was mixed at 1400 rpm for 2 h at 4 °C and centrifuged for 10 mins at 3486 × g at 4 °C to separate the supernatant from the precipitated protein. The clear supernatant was added to a LC-MS vial and centrifuged for 5 mins at 3486 × g at 4 °C. The sample was injected (1 µL) in the chromatographic system using full loop mode (5× overfill).

Lipidomic profiling was conducted using a 2.1 × 100 mm BEH C8 column at 55 °C. Mobile phase A consisted of a 2:1:1 mixture of water:ACN:IPA with 5 mm ammonium acetate, 0.05% acetic acid, and 20 µM phosphoric acid. Mobile phase B consisted of 1:1 ACN:IPA with 5 mM ammonium acetate, 0.05% acetic acid. The initial conditions were 99:1 A:B at a flow rate of 0.6 mL/min. The gradient elution program is shown in Supplementary Table 3.

Supplementary Table 3: RP lipid profiling LC gradient elution program.

LC Gradient					
#	**Time (min)**	**Flow (mL/min)**	**% A**	**% B**	**Curve**
1	Initial	0.6	99.0	1.0	Initial
2	0.10	0.6	99.0	1.0	6
3	2.00	0.6	70.0	30.0	6
4	11.50	0.6	10.0	90.0	6
5	12.00	1.0	0.1	99.9	6
6	12.50	1.0	0.1	99.9	6
7	12.55	0.9	35.0	65.0	6
8	12.65	0.8	70.0	30.0	6
9	12.75	0.7	99.0	1.0	6
10	12.95	0.6	99.0	1.0	6
11	13.25	0.6	99.0	1.0	6

Blood plasma mass spectrometer parameters

Mass spectrometric analysis plasma NIST SRM1950 was performed in positive and negative ionization modes (ESI+ and ESI-). The mass spectrometry parameters were set as follows for urine RP profiling: capillary voltage 1.5 kV (ESI+) and 1 kV (ESI-), sample cone voltage 20 V, source temperature 120°C, desolvation temperature 600°C, desolvation gas flow 1000 L/h, and cone gas flow 150 L/h. Data were collected in centroid mode with a scan range of 50-1200 *m/z* with a scan time of 0.1 s. For mass accuracy, LockSpray mass correction was performed using a 600 pg/µL leucine enkephalin solution (*m/z* 556.2771 in ESI+ and 554.2615 in ESI-) in 1:1 water:ACN solution at a flow rate of 15 µL/min. Lockmass scans were collected every 60 s and averaged over 4 scans. The mass spectrometer was operating in Fast DDA mode. The intensity threshold of the precursor ion was set to 100 K to trigger MS^2 fragmentation that was performed in centroid mode with a scan range of 50-1200 *m/z* and a scan time of 0.25 s. MS^2 was switched back to MS survey function after 2 s acquisition. Deisotoped peak selection option was enabled. The collision energy was set to the ramp of 15–30 eV and 30-60 eV for MS^2 acquisition of low and high mass ions, respectively.

Supplementary References

1. Sumner, L.W. et al. Proposed minimum reporting standards for chemical analysis Chemical Analysis Working Group (CAWG) Metabolomics Standards Initiative (MSI). *Metabolomics* **3**, 211-221 (2007).
2. NIST (https://www.nist.gov/programs-projects/nist23-updates-nist-tandem-and-electron-ionization-spectral-libraries; 2023).
3. Djoumbou Feunang, Y. et al. ClassyFire: automated chemical classification with a comprehensive, computable taxonomy. *Journal of Cheminformatics* **8**, 61 (2016).
4. Albreht, A. et al. Structure elucidation and mitigation of endogenous interferences in LC-MS-based metabolic profiling of urine. *Analytical Chemistry* **94**, 1760-1768 (2022).
5. Lewis, M. et al. An Open Platform for Large Scale LC-MS-Based Metabolomics. (2022).
